# No-ozone cold plasma induces apoptosis in human neuroblastoma cell line via increased intracellular reactive oxygen species (ROS)

**DOI:** 10.1186/s12906-023-04313-0

**Published:** 2024-01-20

**Authors:** Jung-Han Lee, M Shriya Jaiswal, Yoon-Seo Jang, Jeong-Hae Choi, Gyoo-Cheon Kim, Jin-Woo Hong, Dae-Seok Hwang

**Affiliations:** 1 Department of Oral and Maxillofacial Surgery, Dental and Life Science Institute, Dental School, Pusan National University, Busan, South Korea; 2 Department of Research and Development, FEAGLE Corporations, 70-6, Jeungsan-ro, Mulgeum-eup, Yangsan-si, 50614 Gyeongsangnam-do South Korea; 3 Department of Oral Anatomy and Cell Biology, School of Dentistry, Pusan National University, Busan, South Korea; 4 Department of Internal Medicine, School of Korean Medicine, Yangsan Campus of Pusan National University, Beomeo-ri, Mulgeum-eup, Yangsan-si, 50612 Gyeongsangnam-do South Korea; 5 Dental Research Institute, Pusan National University Dental Hospital, Yangsan, South Korea; 6 Department of Oral and Maxillofacial Surgery, School of Dentistry, Pusan National University, Beomeo-ri, Mulgeum-eup, Yangsan-si, 50612 Gyeongsangnam-do South Korea

**Keywords:** No-ozone cold plasma, Plasma activated medium, Anti-cancer effect, Neuroblastoma

## Abstract

**Background:**

This study aimed to evaluate the effect of argon-based No-ozone Cold Plasma (NCP) on neuroblastoma cancer cell apoptosis.

**Methods:**

Experiments were performed with SK-N-SH and HS 68. Cell cultures were treated with NCP for 1, 3, and 5 min. NCP was applied using three different strategies: direct NCP application to cell cultures, to only media, and to only cells. Evaluation of cell viability and the level of the reactive oxygen species (ROS) was performed. N-acetyl-L-cysteine (NAC) was also used to antagonize intracellular ROS. Cleaved caspase 3, PARP, aquaporin (AQP) 3 and 8 were detected.

**Results:**

NCP induced a gradual decrease in the SK-N-SH cell viability. In contrast, the viability of HS 68 cells did not change. SK-N-SH cells viability was reduced the most when the only media-NCP application strategy was employed. Intracellular ROS levels were significantly increased with time. Cleaved caspase 3 and PARP were increased at 6 h after NCP application. SK-N-SH cells remained viable with NAC after NCP application. AQP 3 and 8 were over-expressed in SK-N-SH cells.

**Conclusion:**

These findings demonstrate the anti-cancer effect of NCP on neuroblastoma cells. NCP enhanced the selective apoptosis of neuroblastoma cells due to the increased intracellular ROS.

**Supplementary Information:**

The online version contains supplementary material available at 10.1186/s12906-023-04313-0.

## Introduction

 Neuroblastoma is the most common extracranial solid cancer and the third most common cancer in children after leukemia and brain cancer. This type of cancer accounts for approximately 8% of all childhood cancers under 15 years of age, compromising 15% of the overall childhood cancer mortality [1]. Treatment and outcomes of neuroblastoma depend on the risk group a person is in [2]. Patients with low- or intermediate-risk neuroblastoma have excellent outcomes with minimal therapy [3–5]. However, the prognosis of high-risk stage neuroblastoma is very poor, as the proportion of patients surviving five years is only 34% [5–7]. To improve the cure rate of high-risk neuroblastoma patients, multi-modal treatments, such as traditional surgery, chemotherapy, radiation, and immunotherapy, have been applied [8–11]. Unfortunately, resistance to chemotherapeutic drugs, unresectable masses, complications after radiation treatment, etc. confer a poor prognosis in high-risk neuroblastoma patients [12]. Hence, additional or improved treatment approaches are needed.

Plasma is referred to as the fourth state of matter, which has high reactivity and contains a mixture of electrons, positive and negative ions, radicals, and various excited molecules and atoms [13]. Historically, plasmas could be produced only at high temperatures over 3,000℃ or in a vacuum. However, recent breakthroughs in plasma physics have allowed the development of plasma at room temperature and atmospheric pressure [14].

Plasma medicine is a novel scientific field that has recently emerged as a result of the significant developments in Cold Atmospheric Plasma (CAP). Noble gases, such as argon and helium, are applied to high voltage to create CAP, which in turn enables the creation of stable and predictable plasma. [15]. Plasma sources under well-controlled temperatures below 40℃ have been designed to permit safe plasma applications in human bodies [16, 17]. CAP has already proven to be effective in sterilization, wound healing, hospital hygiene, disinfection, dental care, anti-aging of the skin, and the biocompatibility of implants [18–21].

Recent studies have focused on the potential of CAP to offer an alternative and more effective cancer treatment option. It has already proven its effectiveness in selectively eradicating cancer cells in vitro and decreasing tumor sizes in vivo [22–26]. More specifically, CAP impacts cancer cell apoptosis via reactive oxygen species (ROS) or nitrogen species (RNS), activation of the p53 protein, activation of the p21 CKS inhibitor, and cell cycle arrest. Above all, ROS, a product of CAP, is particularly effective on cancer cells [27, 28]. ROS is a chemical species that includes superoxide anions (O_2_^−^), hydrogen radicals (^°^OH), and hydrogen peroxide (H_2_O_2_) [29]. Especially high concentrations of H_2_O_2_ have been suggested to be the major anti-cancer reactive species that cause apoptosis of cancer cells in vitro [30, 31].

CAP has a selective anti-cancer capacity that tends to resist the growth of cancer cells rather than the growth of normal cells [32]. Homologous cells seem to be tolerable or even resistant to CAP [33, 34]. Consequently, CAP exhibits strong anti-cancer effects on several cancer cell lines, as reported in the literature, such as skin cancer, brain cancer, breast cancer, lung cancer, colorectal cancer, cervical cancer, leukemia, and liver cancer.

In this research, a new plasma device that generates a specific type of plasma was developed and used in the experiment. This device maintains an ozone level of 0.006 ppm at low temperatures (below 30 °C). We named this plasma ‘NCP (No-ozone Cold Plasma)’.

The purpose of this study was to evaluate the effects of NCP on the apoptosis of human neuroblastoma cells. Also, this study investigated the most effective application of NCP on selective apoptosis of human neuroblastoma cells and identified the exact mechanism of response in human neuroblastoma cells.

## Materials and methods

### NCP device

**Fig. 1:**
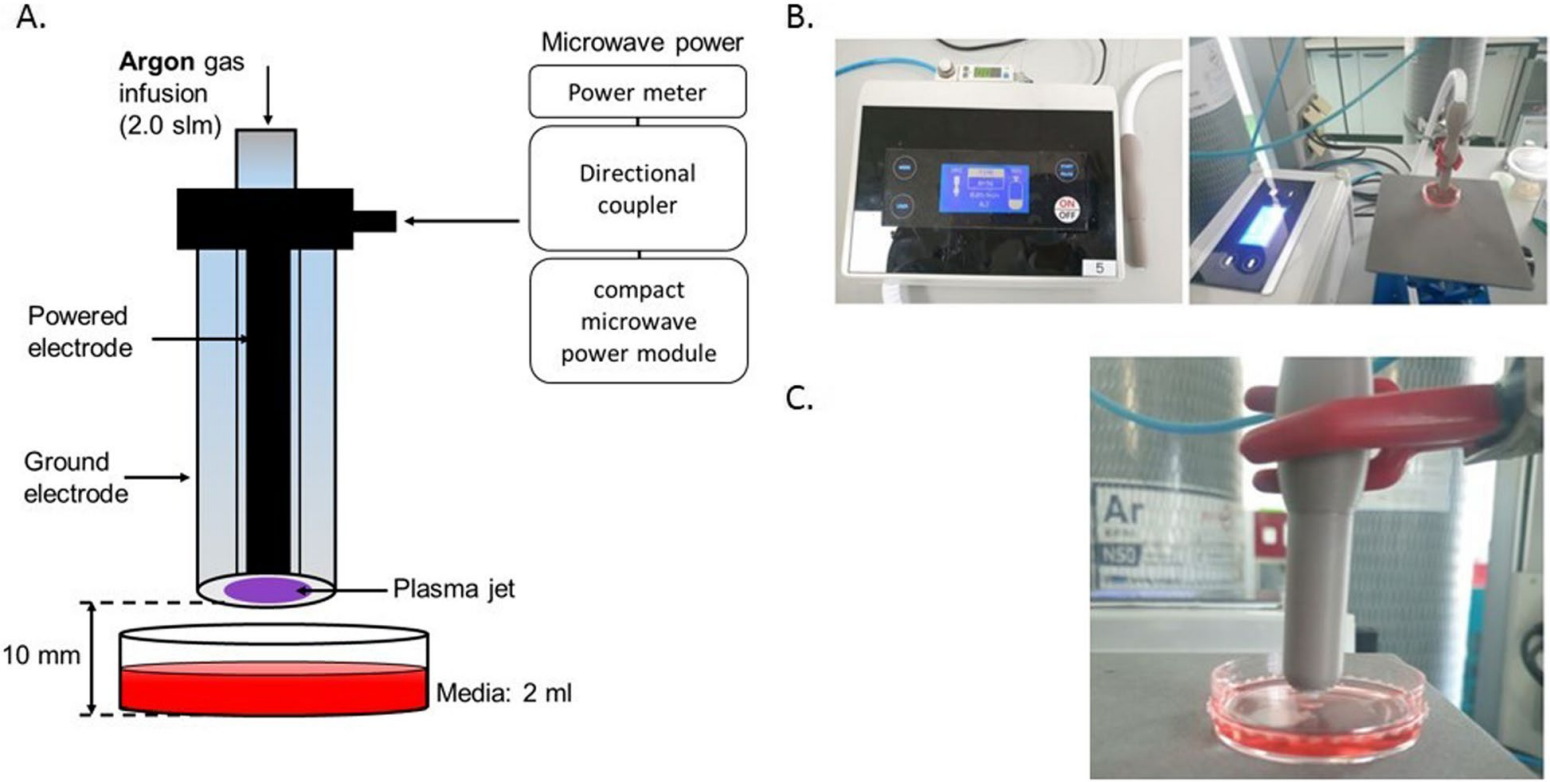
The schematic diagram of NCP-generating device (**A**) and the application of no-ozone cold plasma into the culture dish **(B & C**)

For this study, a dielectric-barrier-discharge-(DBD)-type NCP device developed by FEAGLE Corporation (Yangsan-si, South Korea) was adopted. Argon gas was used as a buffer gas and blown into the plasma source at 2.0 slm (standard liter per minute). NCP was formed by applying a high voltage (3 kV) to the plasma source. A plasma glow was formed within the electrodes, but it did not extend to the end of the electrodes. The temperature of the NCP flow at 1 cm from the electrode end was maintained under 35 °C for 10 min, and no UVs were detected at this condition. Along with that, the value of ozone in this device was found to be 0.006ppm which seems to be much less than the level of ozone recommended by the Food and Drug Administration (FDA) which is 0.05ppm. The distance of the skin from the electrodes was kept at 1 cm [21, 35–37] (Fig. 1).

### Cell culture

Human neuroblastoma cells (SK-N-SH) were purchased from the Korean Cell Line Bank (Seoul, Korea). Non-tumorigenic fibroblast HS 68 (CRL-1635) cells were purchased from the American Type Culture Collection (Manassas, Virginia, USA). Cells were cultured in suitable media (SK-N-SH; RPMI-1640 media, HS 68; DMEM media) with 10% of fetal bovine serum, 100 U/mL penicillin, and 100 U/mL streptomycin at 37 °C in a humidified 5% CO_2_ atmosphere. The cell passage for SK-N-SH and HS 68 subculture were within passage number 3–7.

### NCP device application

The cells were seeded in 35 mm dishes at 2 × 10^5^ cells/ml and incubated for 24 h in a CO_2_ incubator. After culturing the cells for 24 h, NT (plasma non-treat) was treated only with fresh medium without NCP treatment. The NCP 1, 3, and 5-min groups were treated with NCP for 1 min, 3 min, and 5 min, respectively, in the new dishes with fresh media and later it was transferred to the dishes with cells After incubation for 24 h after plasma treatment, all samples were subjected to SRB assay.

### SRB cell viability assay

In the experiment, 24 h after NCP treatment, cells were fixed with 4% PFA for 1 h. The wash process was repeated twice, and staining was performed for 1 h using sulforhodamine B (SRB) dye. After 1 h, the washing process was repeated with 1% acetic acid. When the cell dishes dried completely, photography was performed.

After the photomicrograph, the cells were treated with 10 mM Tris solution to elute the stained SRB dye, and 150 µl of each solution was transferred into 96-well plates. Absorbance was detected using a microplate reader at a wavelength of 515 nm.

SRB assay was performed by incubating cells with N-acetyl-L-cysteine ​​(NAC, 1 mM; Sigma-Aldrich, St. Louis, MO, USA) for 1 h before NCP application for ROS scavenger pre-treatment. All processes were carried out in the same manner as above.

### Western blot analysis

The cells were washed with phosphate buffer saline (PBS) and harvested in ice-cold lysis buffer (containing 50 mM Tris/HCl, pH 7.5, 150 mM NaCl, 1%(v/v) Nonidet P40, 10%(v/v) glycerol, 1 mM PMSF, 1 mM dithiothreitol, 20 mM NaF and 1 mM EDTA, containing a protease inhibitor cocktail (Roche, Basel, Switzerland)). After holding at 4 °C for 30 min, the test tubes were centrifuged at 12,000 rpm and at 4 °C for 20 min. The supernatant was separated and transferred to the new tubes. The total protein content of the lysate was quantified by the Bradford Protein Assay method using the Bio-Rad Protein Assay (Bio-Rad Laboratories, Hercules, CA). Lysate samples (25–35 µg) were mixed with 5X sample buffer and then boiled at 95 °C for 5 min. Lysate samples were resolved by SDS/PAGE (8–15% gel) and transferred to the PVDF membranes (Merck, NJ, USA). The amount of protein expression was analyzed using the Image J program (NIH- https://rsb.info.nih.gov/ij/) and no additional plug-in was used.

After the transfer process, the protein markers displayed on the membrane were checked and cut according to the size of each antibody. Experiments were then conducted with the sliced membrane. The membrane was blocked with 5% skim milk for 1 h at RT. Next, the membrane was probed and analyzed using primary antibodies against cleaved caspase 3, cleaved poly (ADP-ribose) polymerase (PARP), AQP 3 (1:1000; Cell Signaling, Danvers, MA, USA) and AQP 8 (1:200; Santa Cruz Biotechnology, Dallas, TX, USA).

The bands were detected with advanced ECL™ western blotting detection reagents (Amersham Biosciences; Little Chalfont, UK). Finally, loading was verified using the anti-GAPDH antibody (1:5,000; Santa Cruz Biotechnology, Dallas, TX, USA) and beta-actin (1:500; Santa Cruz Biotechnology, Dallas, TX, USA). Images were taken using an Image Quant LAS 4000 (GE, Piscataway, NJ, USA).

### Amplex red assay

To determine the effect of NCP on the oxidation of Amplex Red, a hydrogen peroxidase assay kit was used.

The cells were seeded in a 35 mm dish at 1.25 × 10^5^ cells/ml and cultured in incubation for 24 h. After treatment with NCP-PAM for a non-treat, 1, 3, and 5 min in the culture, and incubated for 0, 2, 4, and 6 h. The concentrations of hydrogen peroxide, nitrite, and the total nitrite plus nitrate were determined using a colorimetric assay kits-hydrogen peroxidase assay kit (Cat. No. A22188, Invitrogen, CA, USA) and an in vitro nitric oxide (nitrite/nitrate) assay kit (Cat. No. STA-802, Cell Biolabs, Inc., CA, USA) following the manufacturer’s instructions. The same protocol was used for the experiment with the cells.

Each sample was treated with 50 µL of Amplex® Red reagent/HRP working solution for 30 min, and absorbance was detected and measured using a microplate reader at a wavelength of 560 nm.

### DCF-DA assay

For visualization and analysis of intracellular ROS, the oxidation-sensitive probe DCF-DA (thermo fisher scientific, MA, USA) was used.

At first, the cells are seeded in a 96-well plate at 1 × 10^4^ cells/well and incubated for 24 h. After that, SK-N-SH cells were exposed to a 5-µM DCF-DA solution, 150 µl for each well plate, and cells were incubated for 30 min at 37˚C in the dark. After the cells were washed with PBS three times, the NCP was then treated with 100 µl of PAM. DCF fluorescence was observed using fluorescence microscopy and was quantified by a fluorescence multi-well plate reader (BioTek, Highland Park, VT, USA) with an excitation wavelength of 490 nm and an emission wavelength of 535 nm.

### Data analysis

Data are presented as the mean ± standard deviation of the mean (SEM) of at least three independent experiments. All statistical analysis was performed using Microsoft Excel (*Microsoft2013, Redmond, WA, USA*) and IBM SPSS ver.26 (*IBM, USA*). The two-tailed Student’s t-test and one-way and two-way ANOVA test were performed to assess the statistical significance for differences in mean values, and the significance was set at *p* < 0.05. SEM, or standard error of mean, was applied to the data. A post-hoc test was performed to indicate a significant result for each groups.

## Results

### NCP decreased neuroblastoma cell viability

**Fig. 2:**
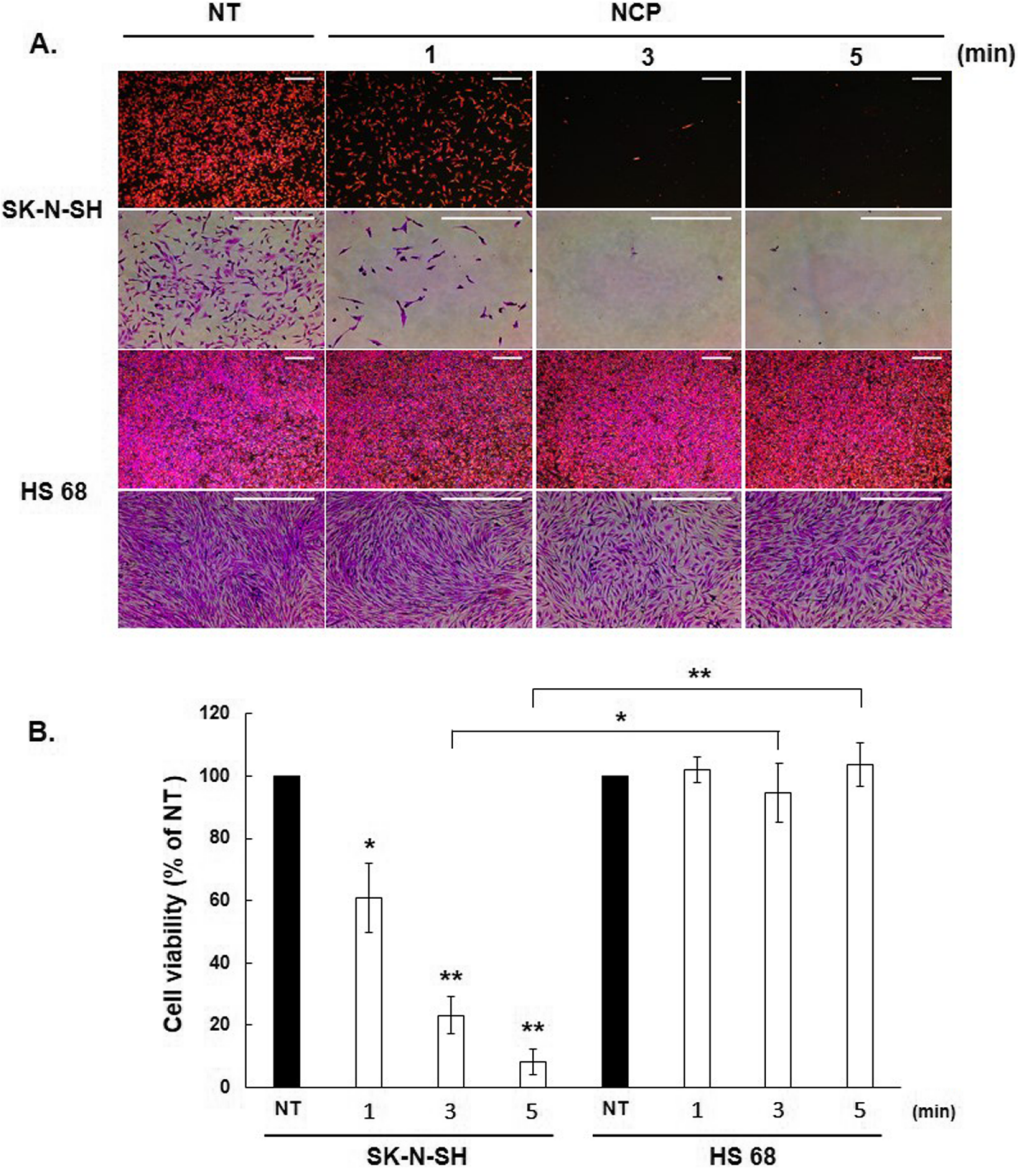
The effect of NCP on cell viability of the human neuroblastoma cell line (SK-N-SH) and human fibroblast cell line (HS 68). **A**. The human neuroblastoma cell line (SK-N-SH) and human fibroblast cell line (HS 68) were treated with NCP by direct treatment (DT) method and SRB assay was performed. Each group was treated with NCP except NT (plasma non-treat) for 1 min, 3 min, and 5 min. SRB assay was performed 24 hours after NCP treatment. **B**. The results of the SRB assay were digitized and presented as a graph. *indicates *p* < 0.05, ** *p* < 0.01

To determine the effect of NCP treatment on the viability of neuroblastoma cells in vitro, the SRB assay was performed. At first, the human neuroblastoma cell line (SK-N-SH) and human fibroblast cell line (HS 68) were prepared. Cells were treated with the DT (Direct Treat) method for NT (plasma non-treat), 1, 3, and 5 min with the NCP device as described above. SK-N-SH neuroblastoma cell line showed a statistically significant gradual decrease in its cell viability over time under NCP treatment. In contrast, the HS 68 fibroblast cell line maintained its cell viability after NCP treatment (Fig. 2). When the significance between the two groups (SK-N-SH cell, HS68 cell) was checked, the cell viability of SK-N-SH cells was significantly reduced when NCP was treated for 3 and 5 min. In particular, when NCP was treated for 5 min, there was a highly significant difference between the two cells.

**Fig. 3:**
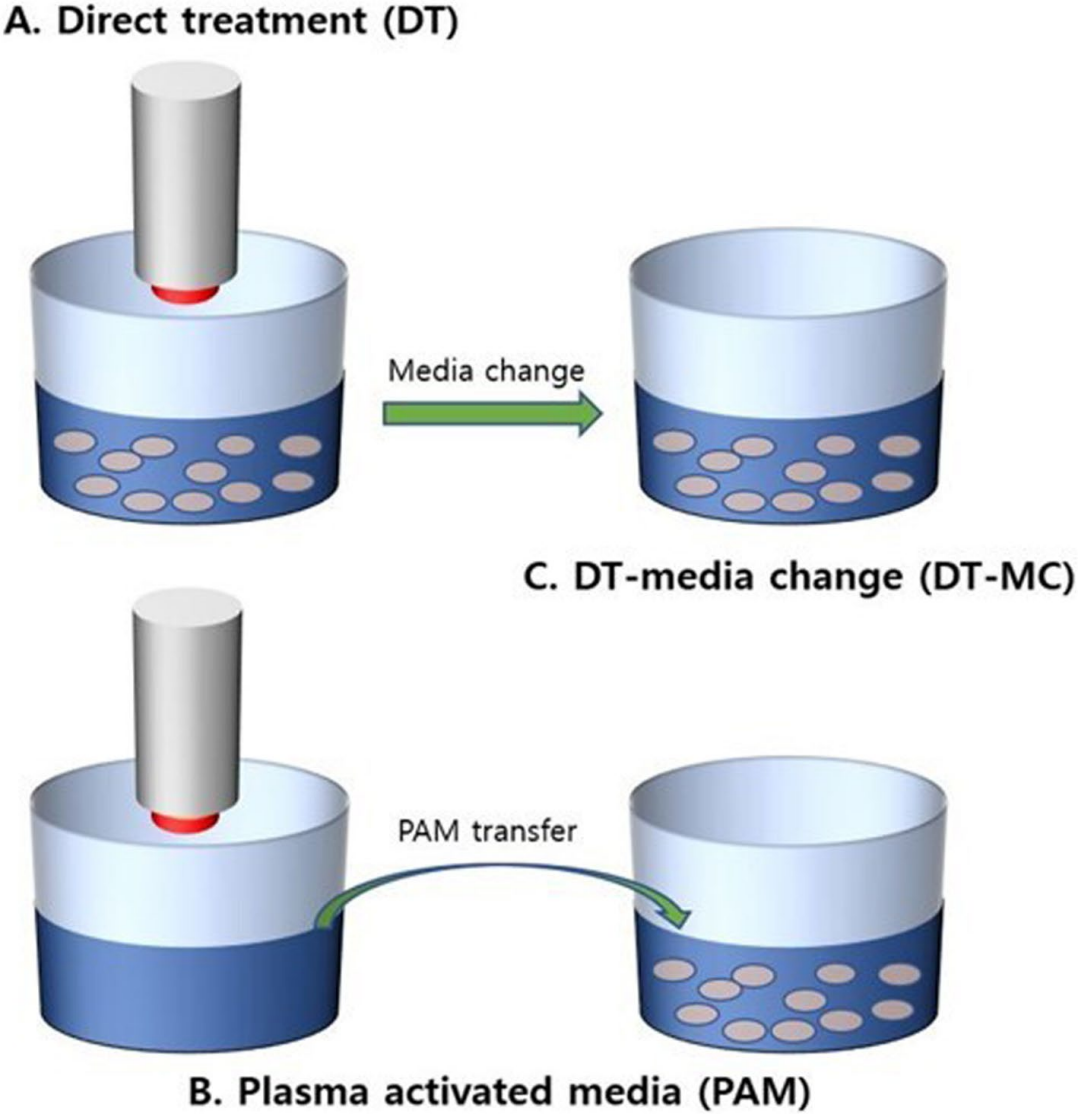
Application of NCP-generating device with three different strategies. **A**. Direct application of NCP to cells seeded in dishes (DT). **B**. NCP application to media before cells are seeded and plasma-activated medium applied to cells (PAM). **C**. Change with fresh media after direct NCP application to cells (DT-MC)

**Fig. 4:**
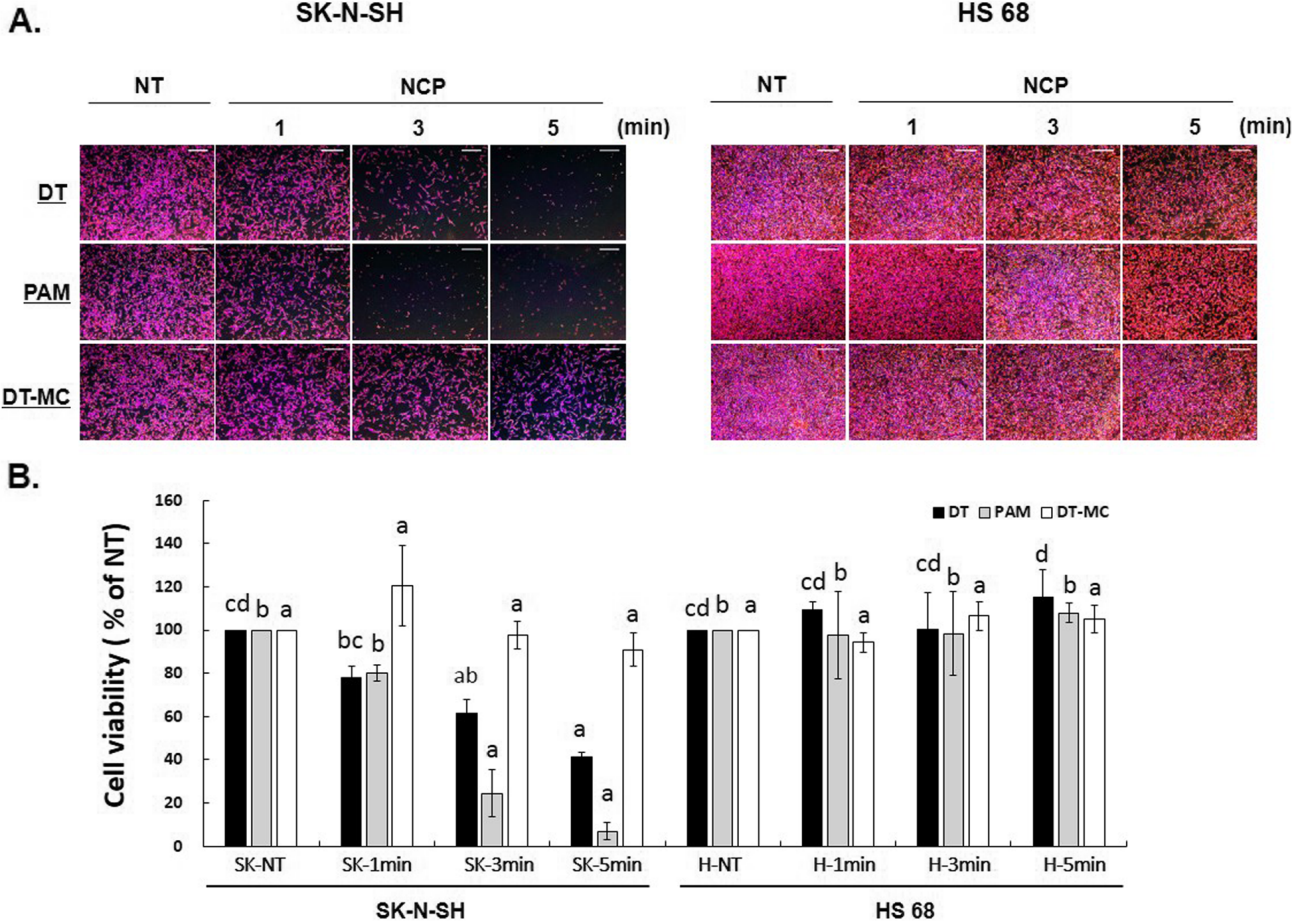
Cell viability was confirmed when SK-N-SH cells and HS 68 cells were treated with NCP in various ways. **A**. The human neuroblastoma cell line (SK-N-SH) and human fibroblast cell line (HS 68) were treated with NCP in three ways. According to each method, NT (plasma non-treat) and NCP were treated for 1 min, 3 min, and 5 min, and then cultured for 24 h. DT: direct NCP treatment, PAM: plasma activated medium, DT-MC: direct NCP treatment, and media change. **B**. The results of the SRB assay were digitized and presented as a graph. Vertical axis: (% of NT). * Different letters (a, b, c, d) indicate statistically significant differences by one-way ANOVA (*p* < 0.05). The bars in this graph that have the same letters, the groups are not significant among themselves. But the bars which have different letters are statistically significant among themselves

Application of the NCP device using the three strategies was also examined (Fig. 3). SRB assay was conducted in three ways. Direct NCP treatment (DT) and plasma-activated medium (PAM) application showed a significant decrease in cell viability with the increase in the treatment time of NCP. In particular, the 5-min PAM treatment showed a high statistical significance in reducing cell viability (Fig. 4).

Also, when a statistical significance analysis was performed, there was a significant difference in the NT group of DT treatment of both cells. When compared with HS68 cells of the DT treatment group, the SK-N-SH cells of the 3-min and 5-min groups showed statistical significance. In addition, the treatment of PAM was highly significant when SK-N-SH cells were treated for 3 and 5 min. DT-MC treatment group did not differ significantly in both cells (Fig. 4b).

A substantial reduction in 5-min PAM treatment for SK-N-SH cells was observed between the DT, PAM, and DT-MC groups. It was confirmed that there was a significant difference in the 5-min treatment between the PAM and DT-MC groups. In addition, the NCP processing method in the SK-N-SH cell and HS68 cell was compared. Significance was confirmed when DT was treated for 5 min, and PAM treatment was confirmed to significantly reduce the cells at 3 and 5 min in both cells. (Fig. 4). In the subsequent experiment, the NCP was treated with the PAM method for 5 min. A post-hoc test was performed to indicate a significant result for each group. All data were analyzed using two-way ANOVA followed by post hoc test for multiple comparisons. Different letters (a, b, c, d) indicate statistically significant differences by two-way ANOVA (*p* < 0.05). The bars in this graph that have the same letters, the groups are not significant among themselves. However, the bars which have different letters are statistically significant among themselves. For instance, DT group of SK-NT and SK-1 min (have same letter “c”) are not significant. However, DT group of SK-NT is statistically different from all other groups of SK-NT, 1 min, 3 and 5 min.

### Western blot analysis after NCP-PAM treatment on SK-N-SH neuroblastoma cells

**Fig. 5:**
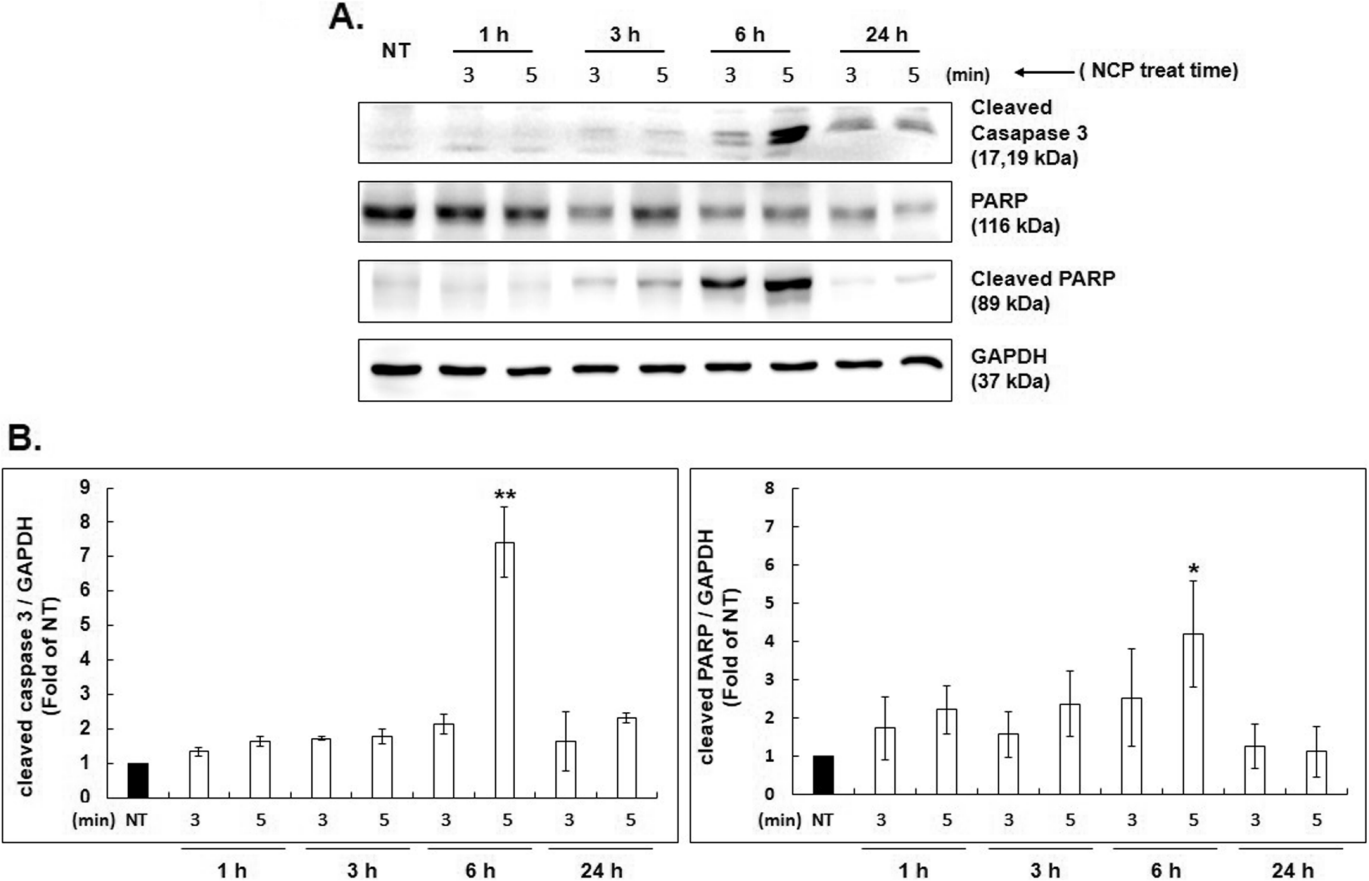
NCP was treated with PAM induces apoptosis of SK-N-SH through the cleaving of caspase3 and PARP. **A**. Western blot analysis of PARP and cleaved caspase 3 showed that SK-N-SH cells were treated with plasma for NT (plasma non-treat), 3 min, and 5 min, and then later for 1 h, 3 h, 6 h, and 24 h. Western blot was performed. GAPDH was used as a loading control for Western blot analysis. **B**. Quantification of the protein was performed to evaluate the effects of NCP-PAM treatment in SK-N-SH cells. Vertical axis: fold of NT. * indicates *p* < 0.05, ** indicates *p* < 0.01

In the previous experiment, it was confirmed that the treatment of SK-N-SH cells with NCP reduced cell viability. A Western blot was performed to confirm whether these findings could lead to apoptosis and cell death. The proteolytic activation of cleaved caspase 3 and cleaved PARP was investigated using western blot analysis to determine SK-N-SH neuroblastoma cell apoptosis after NCP treatment. (Figure. 5) After treating, NT (Non-treat) and NCP for 3 and 5 min, the protein expression was confirmed after 1, 3, 6, and 24 h. PAM was used as a treatment method.

(A) Expression of cleaved caspase 3 was increased the most after 6 h of treatment with NCP-PAM for 3 and 5 min. In particular, it was confirmed that the expression of cleaved caspase 3 increased the most when 6 h had elapsed after treatment with NCP-PAM for 5 min, compared to NT. Similarly, cleaved PARP (89 kDa) appeared to increase gradually at 3 and 6 h after treatment with NCP for 3 and 5 min. In particular, cleaved PARP expression increased the most at 6 h after NCP-PAM treatment for the 5 min group.
(B) The protein expressions of cleaved caspase 3 and cleaved PARP (the results of the above western blotting) were quantified graphically using GAPDH.

The results showed that, when compared to NT, the protein expression of cleaved caspase 3 (1, 3, 6, and 24 h) following treatment with NCP-PAM for 3 and 5 min was the most significant after 6 h. It was confirmed that the expression had increased. In addition, it was confirmed that the expression of cleaved PARP was most significantly increased after 6 h of treatment with NCP-PAM for 5 min compared to NT.

### Alterations of H_2_O_2_ level in media after NCP-PAM treatment

**Fig. 6:**
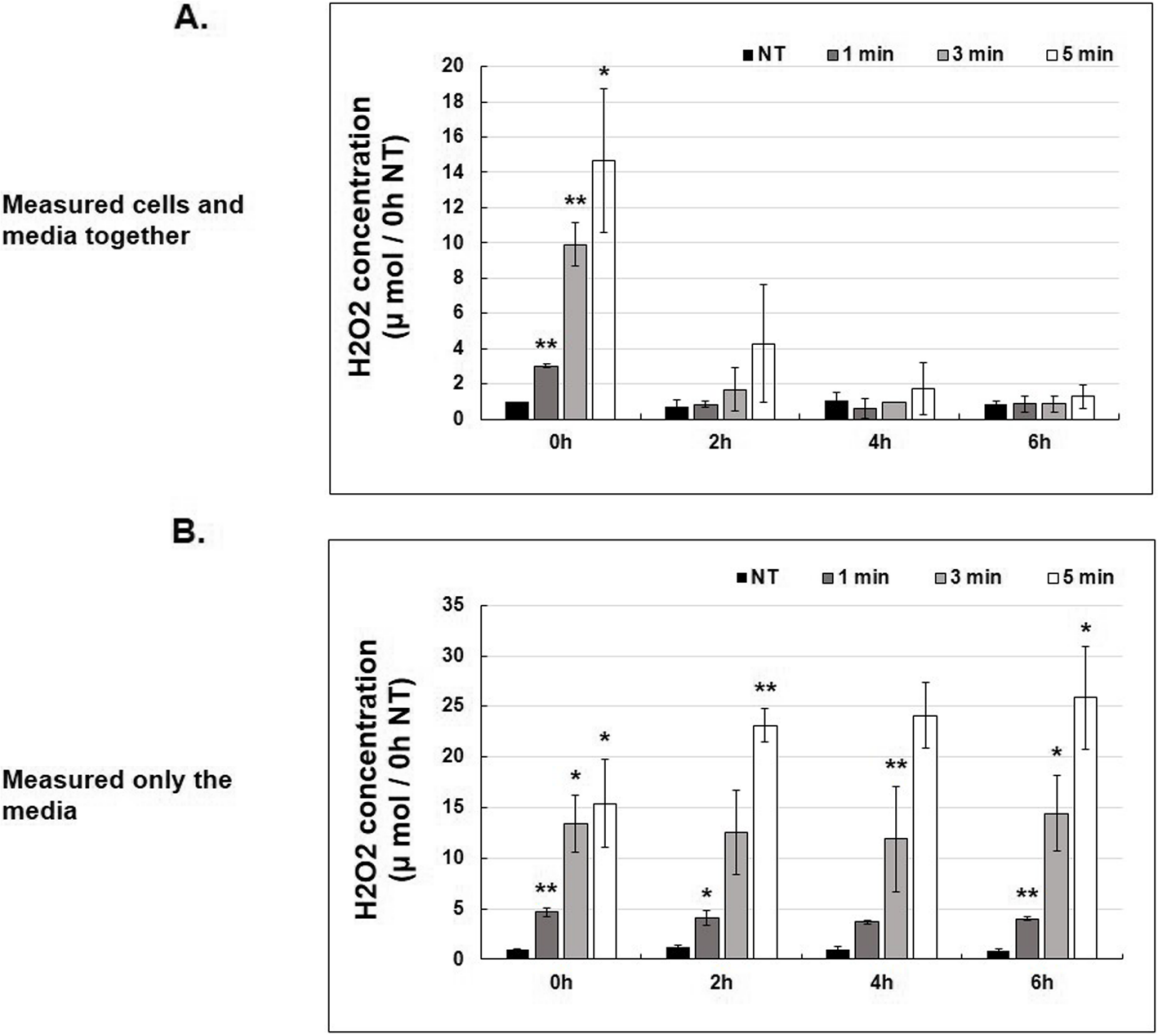
The change of H_2_O_2_ concentration according to NCP-PAM treatment with and without SK-N-SH cells. **A**. In the presence of SK-N-SH cells, NCP was treated with PAM for plasma non-treat, 1 min, 3 min, and 5 min and cultured for 0 h (immediately after treatment), 2 h, 4 h, and 6 h. Afterward, the concentration of H_2_O_2_ was confirmed. **B**. In the absence of SK-N-SH cells, NCP was treated with plasma non-treat, 1 min, 3 min, and 5 min in the medium for 0 h (immediately after treatment), 2 h, 4 h, and 6 h. Afterward, the concentration of H_2_O_2_ was confirmed. * *p* < 0.05, **  *p* < 0.01

The H_2_O_2_ level in the media was measured using the Amplex red assay to look into the mechanism of cell apoptosis in the SK-N-SH cells following NCP-PAM treatment. Media on dishes with or without seeding of the SK-N-SH cells were prepared and treated with NCP-PAM for NT (Non-treat), 1, 3, and 5 min. Immediately after NCP-PAM treatment, the concentration of H_2_O_2_ in the media increased gradually, depending on the duration of the NCP-PAM treatment. However, at 2 h post-NCP-PAM treatment, the level of H_2_O_2_ in media with SK-N-SH cells was greatly reduced, and as 4 and 6 h passed, it was reduced to a level similar to that of the NT (plasma non-treat) group. However, for the H_2_O_2_ group in the medium without SK-N-SH cells, the H_2_O_2_ concentration was maintained for up to 6 h in the medium and the concentration increased according to the treatment times of 1, 3, and 5 min with NCP-PAM. (Figure. 6)

### NCP-PAM treatment increased the intracellular ROS level in SK-N-SH cells for a certain period

To examine the effect of NCP-PAM treatment on neuroblastoma cell apoptosis, it was necessary to evaluate intracellular oxidative stress. Intracellular ROS levels were examined under the DCF-DA assay.

SK-N-SH cells and HS68 cells were treated with NT (Non-treat) and NCP-PAM for 1, 3, and 5 min, respectively, and changes in ROS levels immediately after treatment, 0.5, 2, 4, and 6 h later, were observed. A one-way ANOVA Tukey post-hoc test was performed to compare both groups. In SK-N-SH cells, 30 min after NCP-PAM treatment, compared to immediately after NCP-PAM treatment, the ROS level increased about 1.5 times (0.5 h group) when compared to NT. There were no significant changes between the cells at 0.5 h.

However, 2 h after NCP-PAM treatment, SK-N-SH cells showed a gradual increase in ROS levels compared to NT cells. In HS68 cells, the ROS change between the groups was not significant whereas there was a significant difference between the two cells in NCP-PAM 5-min groups. Moreover, there was a significant difference between SK-NT and SK-3 min; SK-3 min and HS-NT at 2 h.

When 4 h passed after NCP-PAM treatment, the ROS levels significantly increased in SK-N-SH cells when compared to NT with 1 min, 3 min, and 5 min groups.

**Fig. 7:**
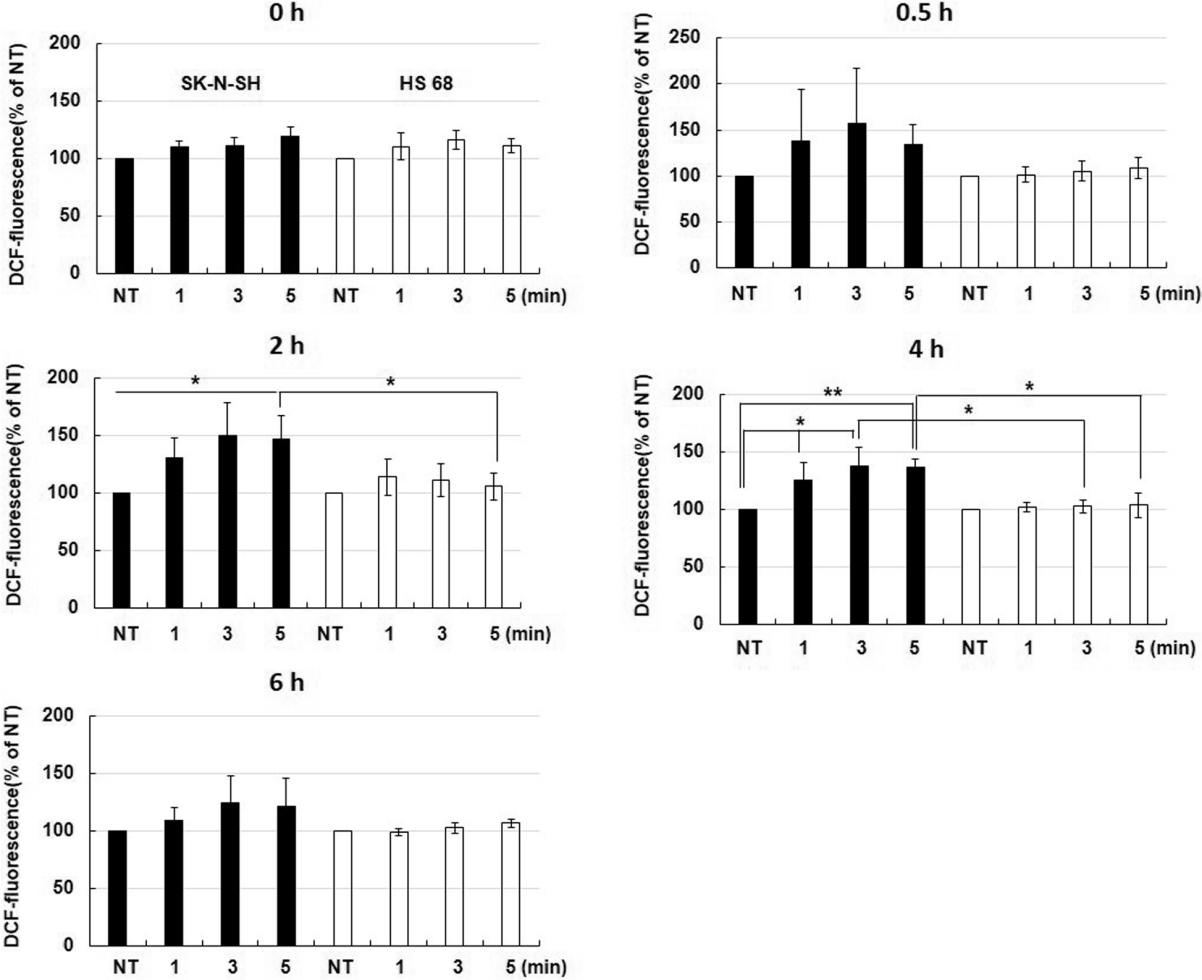
SK-N-SH cells and HS68 cells were treated with NCP-PAM and the change in intracellular ROS level was confirmed. SK-N-SH cells and HS68 cells were treated with NCP-PAM for plasma non-treat, 1 min, 3 min, and 5 min, and incubated for 0 h (immediately after treatment), 0.5 h, 2 h, 4 h, 6 h, and then intracellular ROS level was measured. (Black bar=SK-N-SH cell, White bar=HS68 cell). One way ANOVA tukey post-hoc test was performed to compare both groups. * indicates *p* < 0.05, ** indicates *p* < 0.01

There was no difference in ROS level between the groups in HS68 cells, and there was a significant difference in ROS level when comparing the two cells. At 6 h after NCP-PAM treatment, it was confirmed that the ROS level of SK-N-SH cells was reduced immediately after treatment (Figure. 7).

### Intracellular ROS scavenger partially antagonizes cell apoptosis after NCP-PAM treatment

To clarify the effect of ROS on SK-N-SH cell apoptosis, treatment of SK-N-SH cells with NCP-PAM and SRB to confirm cell viability using a ROS scavenger (N-acetyl-L-cysteine: NAC) was applied. A Western blot was performed to confirm the change in assay and protein expression. NCP was applied to SK-N-SH cells using NT, PAM, DT, and DT-MC.

SK-N-SH cells were treated with NCP by NT, PAM, DT, and DT-MC, and cell viability was confirmed in NAC-NT (group not treated with NCP after NAC treatment) and NAC-PAM (treated with PAM after NAC treatment) group.

(A)Intracellular ROS scavengers (NAC) decreased cell viability by 75.24% when treated with PAM and DT compared to NT as in the previous results. However, looking at the cell viability of the NAC + PAM group, the cell viability was increased compared to the group treated with PAM alone, and the cell viability decreased by about 40.96% compared to the NT group. So, we can say that the treatment with NAC and PAM partially antagonizes cell apoptosis after NCP treatment. This showed a significant difference.

(B) SK-N-SH cells treated with NCP by the methods of NT, PAM, and DT, NAC-NT (group treated with NAC and not treated with NCP), and NAC + PAM (treated with NAC and then PAM) and the protein expression of cleaved caspase3 and PARP was observed using Western blotting.

As in the previous results, NT, PAM, and DT groups confirmed cleaved caspase3 and PARP. Surprisingly, in the NAC-treated group, no cleaved form was observed in NAC-NT, but in the NAC + PAM group, cleaved forms were observed in caspase3 and PARP.

When protein expression was quantified graphically, the group treated with PAM and DT increased the expression of cleaved caspase3 by up to 4 times when compared to NT, and in the NAC + PAM group also increased by 2 times when compared to NAC-NT.

**Fig. 8:**
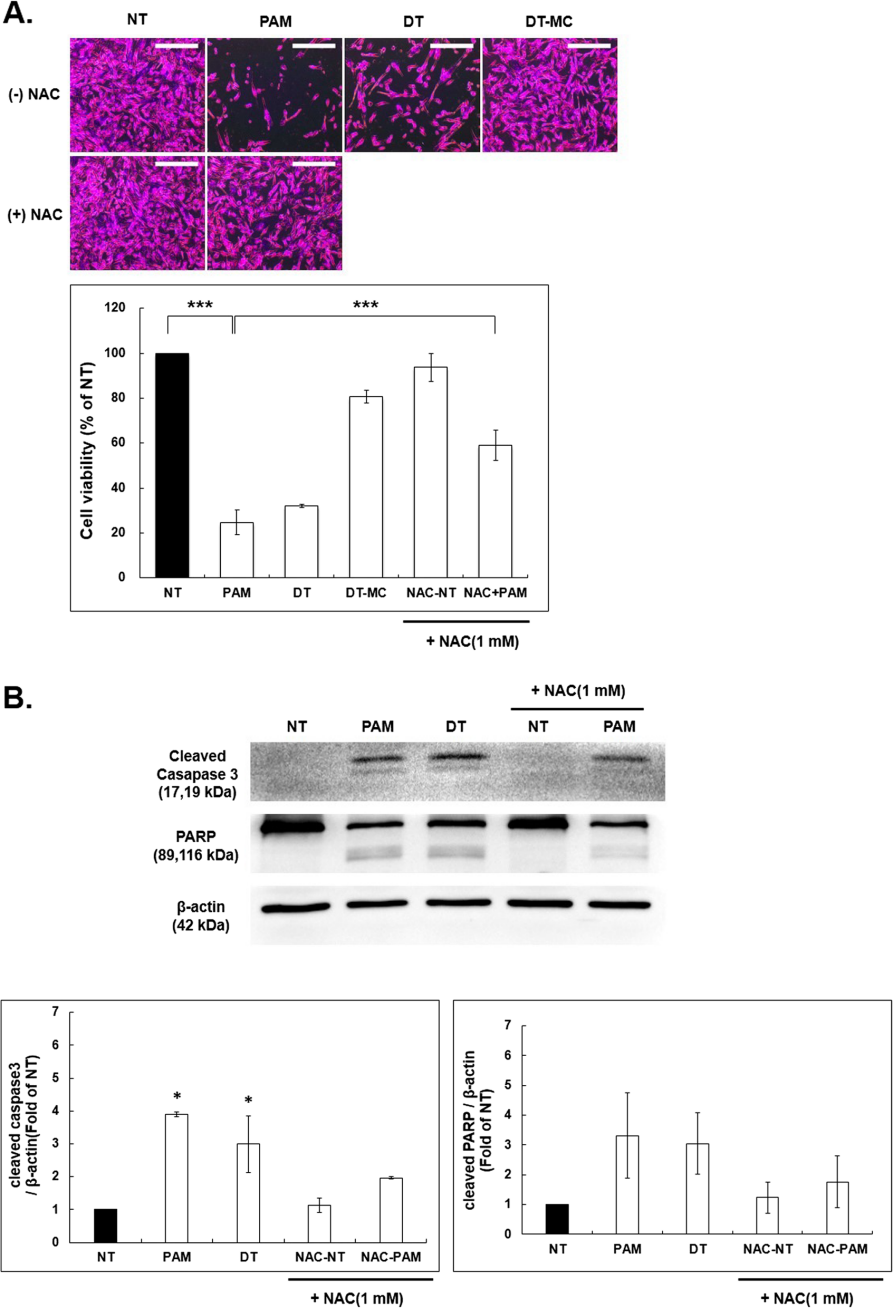
**A**. Effect of NCP on cell viability of SK-N-SH cells using ROS scavenger. NCP was added to SK-N-SH by plasma non-treat, PAM (plasma activated media), DT (direct plasma treat), DT-MC (direct plasma treat - media change) methods, and NAC (ROS scavenger)-NT (PAM non-treat after NAC treatment) treat) and NAC-PAM (NAC-treated followed by PAM-treated) for SRB assay. All NCP treatments were performed for 5 min. NCP was cultured for 24 h after treatment, NAC 1mM was pretreated for 1 h before PAM treatment. SRB assay was quantified and presented as a graph. *** *p* < 0.001. **B**. Western blotting was performed with the same group and experimental conditions as in **A**. The results were quantified and presented as graphs. Vertical axis: fold of NT. * *p* < 0.05

The protein expression of cleaved PARP was also increased up to 3.5 times in the group treated with PAM and DT alone compared to NT, and the expression of cleaved PARP in the NAC + PAM group increased by 1.8 times compared to NAC-NT. (Fig. 8)

### Expression of aquaporin (AQP) in the cytoplasmic membrane which causes transmembrane diffusion of H_2_O_2_

**Fig. 9:**
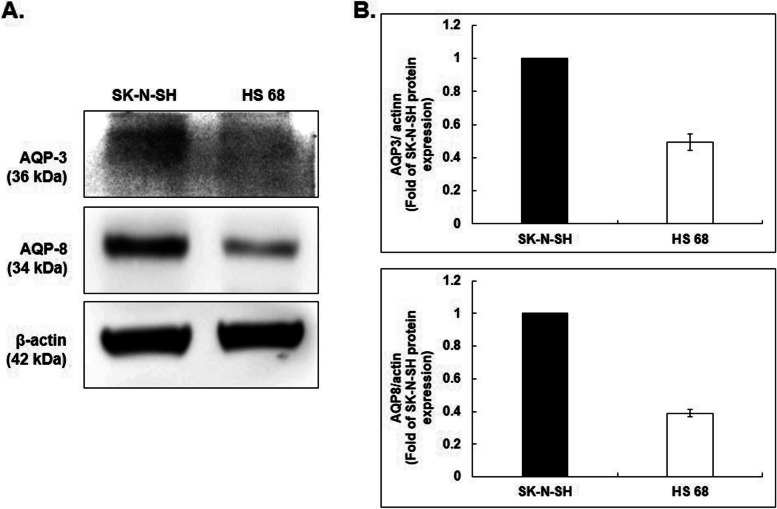
Confirmation of Aquaporin (AQP) expression on the cytoplasmic membrane of SK-N-SH cells and HS 68 cells. **A**. The expression of AQP (aquaporin) 3 and AQP8 in SK-N-SH cells and HS68 cells was confirmed by Western blot β-actin was used as a loading control for Western blot analysis. **B**. As a result of Western blot, the expression levels of AQP3 and AQP8 were quantified with β-actin and shown in a graph. Vertical axis: fold of SK-N-SH cell protein expression

Aquaporins 3 and 8 are involved in the transmembrane diffusion of H_2_O_2_. Therefore, without the use of NCP treatment, a difference was observed in the proteolytic activation of AQP 3 and 8 between SK-N-SH neuroblastoma cells and HS 68 fibroblast cells. (A) Both AQP 3 and 8 were significantly over-expressed in SK-N-SH cells compared to the HS 68 cells. (B) As a result of quantitative analysis with β-actin, the protein expression of AQP 3 and 8 was significantly increased in SK-N-SH neuroblastoma cells. (Figure. 9)

## Discussion

Recently, the development of innovative CAP techniques has underlined the potential applications of this method in biology and medicine. [38, 39]. Depending on their configuration, they can be used in several biomedical approaches [18–21, 39]. Furthermore, it offers clinicians a unique atraumatic approach to the surrounding living tissue method that can be safely applied to patients [16, 17]. ]Since the devices generating DBD plasma have become more compact and lighter in recent years, it has become more convenient to use them for in vitro research. The CAP treatment protocol has been previously described [21, 40].

Several CAP applications were introduced for cancer treatment in 2004 (in vitro) and 2011 (in vivo). Although the efficacy of CAP in other types of cancer has been established, there is no proof of the its effects in the treatment of neuroblastoma [41].

Research has been focusing on activated reactive oxygen species (ROS) and reactive nitrogen species (RNS) due to their anti-cancer effects [42]. H_2_O_2_ has been identified as the primary reactive species among the several CAP-originated ROS species that induce cancer cell death in vitro. [30, 31].

The most important factor in the development of CAP approaches to cancer treatment is cell selectivity. Normal cells require a much higher dosage of CAP to damage normal cells due to their more advanced defense mechanisms [33, 34]. In addition, Keidar et al. found that the dissipated heat released by CAP does not harm normal cells [31]. However, the application of CAP induced apoptosis on cancer cells while normal host cells remained viable and unharmed. Recently, we replaced the term CAP with NCP (No-ozone Cold Plasma). The reason is the use of a different device that emits less ozone from the device (lesser than the recommended value by FDA i.e. 0.05ppm).

The SRB assay cell viability test used in this study demonstrated that the SK-N-SH neuroblastoma cell line exhibited a higher loss in cell viability following NCP (No-ozone Cold Plasma) treatment compared to the HS 68 fibroblast cell line. In contrast, NCP treatment did not induce any differences in the viability of the HS 68 fibroblast cell line. Above all, the SK-N-SH cell line exhibited the most sensitive results. These findings suggest that NCP has a more selective anti-cancer effect on neuroblastoma cells compared to human fibroblast cells. However, further studies need to be carried out with multiple neuroblastoma cell lines.

According to the results obtained from three different NCP application strategies, PAM showed high significance in terms of reducing the viability of SK-N-SH cells. While previous studies primarily preferred the direct NCP application for cancer treatment, the PAM method has become an emerging and hot topic over the past five years due to its prominent advantages: (1) PAM can be stored in the refrigerator and maintain its anti-cancer capacity for at least 7 days. (2) The CAP jet may not easily penetrate skin or tissues, but PAM can be injected into tissue [43, 44]. Moreover, PAM, which has a high concentration of reactive species through chemical or physical means may be a more effective approach to maximize the treatment of cancer. [45, 46]. Therefore, for this study, we used PAM.

To determine the effects of varying concentrations of NCP-PAM originated ROS species, the Amplex red assay and the DCF-DA assay were employed. A significantly increased level of H_2_O_2_ concentration in media was detected immediately after NCP-PAM treatment, and this effect appears to increase with time. However, after two hours of NCP-PAM treatment, the amount of activated H_2_O_2_ in media containing the SK-N-SH neuroblastoma cell line rapidly decreased. In SK-N-SH cells, 30 min after NCP-PAM treatment, compared to immediately after NCP-PAM treatment, the ROS level increased about 1.5 times (0.5 h group) when compared to NT. There was no significance between the cells at 0.5 h. Therefore, it can be suggested that the H_2_O_2_ concentration in media activated by NCP-PAM treatment is connected to the increasing intracellular ROS level, which is, in turn, caused by transmembrane diffusion of NCP-originated ROS species. This hypothesis is supported by the results of this study, where the addition of a ROS scavenger (NAC) in the media in which SK-N-SH neuroblastoma cells were immersed revealed that neuroblastoma cells viability reduced after NCP-PAM application.

AQPs were first discovered as water channel proteins found in the plasma cell membranes of various cells [47]. There are 14 AQPs in mammals, and overexpression of AQPs in tumor cells is generally reported [48]. Among the AQP family, recent research has shown that AQP 1, 3, 8, 9, and 11 are the membrane channels related to H_2_O_2_ transmembrane diffusion [49–53]. Particularly, this study found that AQP 3 and 8 were over-expressed in SK-N-SH cells compared to HS 68 cells, and they seemed to facilitate the H_2_O_2_ transmembrane diffusion. As we have described, induction of ROS stress in SK-N-SH cells is one of the key factors of NCP on NCP-mediated cytotoxicity, the results of Fig. 9 can be one of the explanations of NCP’s selective cytotoxicity against Neuroblastoma.

However, we did not observe the effect of NCP in the presence of aquaporin blockers. However, according to Yan et al., both the silencing of aquaporin 8 and the use of the silver atom, a blocker of aquaporin 8, inhibited the anti-cancer activity of PAM in U87MG glioblastoma cells [54]. Likewise, we think that the use of specific blockers of aquaporins might reduce the anti-cancer activity of NCP.

Extensive research has delved into the fundamental characteristics and mechanisms of apoptosis in mammalian cells. The activation of death receptors, such as tumor necrosis factor receptor (TNFR), and the subsequent recruitment of death-inducing signaling complex (DISC), which cleaves caspase-8 zymogen and further activates effector caspase-3/7, are the usual causes of apoptosis in the context of the extrinsic apoptosis pathway. On the other hand, the death receptor- and mitochondria-mediated apoptotic processes form the basis of the intrinsic apoptosis pathway [55, 56]. Many studies have shown that cancer cells treated with CAP undergo apoptosis. Reactive species that are CAP-originated, especially ROS, cause apoptosis [57]. The rise of intracellular ROS in cancer cells can induce DNA damage and cell apoptosis. Although some studies demonstrated caspase-independent apoptosis in the cancer cells treated with CAP, the majority of CAP-triggered cancer cells died via caspase-dependent apoptosis pathways [56]. ROS species can induce caspase activation and apoptosis via the mitochondrial pathway [58]. After CAP treatment in cancer cells, a mitochondrion-based apoptosis pathway triggered by DNA and mitochondrial damage has been mostly observed [41]. The western blot analysis performed in this study detected high expression of cleaved caspase 3 and PARP after 6 h for the 5-min NCP-PAM treatment group when compared to the NT group of SK-N-SH cells. Therefore, NCP-PAM treatment seems to have a distinct impact on SK-N-SH neuroblastoma cell apoptosis. But as we know, cleaved caspase 3 and PARP are also found downstream of the mitochondrial (intrinsic) pathway, which is similar to other studies. Moreover, our results of Western Blot against PARP-1 and caspase 3 can also be proof of apoptotic cell death. Therefore, it can be said that the mitochondrial pathway can be one of the reason to mediate NCP-induced apoptosis in neuroblastoma [59]. Nevertheless, extrinsic pathway-mediated apoptosis in SK-N-SH cells remains unknown. Therefore, additional research is needed to confirm this complex anti-cancer mechanism through both intrinsic and extrinsic pathways.

CAP has been investigated extensively in cancer treatment recently. In previous studies, CAP treatment was reported as a favorable anti-cancer approach in various cancer cell lines and tumors in animal models [41]. However, our current knowledge and understanding of the anti-cancer mechanism are still very limited. This in vitro study revealed the cancer cell apoptosis of neuroblastoma under NCP application and its anti-cancer mechanism according to ROS. Further research is needed concerning additional reactive species and components generated in NCP. More experimental designs and in vivo studies are needed to elucidate the exact anti-cancer mechanism of NCP on other neuroblastoma cell lines. In the future, standardized or superior NCP devices and NCP treatment strategies will be necessary to facilitate more efficient clinical applications.

## Conclusion

In this study, NCP application to neuroblastoma cell lines showed that:

1. NCP selectively decreased neuroblastoma cell viability and induced apoptosis without harming homologous cells. PAM showed its high efficacy among different treatment strategies.
2. NCP-PAM induced a dose-dependent increase of H_2_O_2_ concentration in media, which is related to the production of ROS. Greater intracellular ROS diffusion was detected in neuroblastoma cells compared to homologous cells after 2 h of NCP-PAM application. In western blot, expression of cleaved caspase 3 and cleaved PARP increased after 6 h post-NCP-PAM application. It can be seen that NCP causes oxidative stress that induces neuroblastoma cell apoptosis. More clearly, with the NAC intracellular ROS scavenger, neuroblastoma cell viability declines after NCP application.
3. Intracellular diffusion of ROS species should be considered among the important factors in neuroblastoma cell apoptosis, as it showed about a 40.96% reduction in PAM-mediated cell death. Neuroblastoma cells exhibited an increased expression of proteolytic activation of AQP 3 and AQP 8 compared to homologous cells, which is related to the transmembrane diffusion of ROS.

This study highlights the novel and potential treatment modality of NCP in neuroblastoma treatment.

### Supplementary Information

**Additional file 1.**

## Data Availability

The dataset supporting the conclusions of this article is included within the article and as a Supplementary files.

## Abbreviations

CAP: Cold Atmospheric Plasma
NCP: No-ozone Cold Plasma
NT: Plasma Non-Treat
DT: Direct Treat
DT-MC: Direct Treat-Media Change
PAM: Plasma Activated Media
DBD: Di-electric Barrier Discharge
