# Supplementary material for: No-ozone cold plasma induces apoptosis in human neuroblastoma cell line via increased intracellular reactive oxygen species (ROS)
Source: BMC Complement Med Ther. 2024 Jan 20;24:46. doi: 10.1186/s12906-023-04313-0 (PMC10799363; doi:10.1186/s12906-023-04313-0)

This is the original western blot data.

The "original", "protein marker" and "original + protein marker merge" files of each antibody are mentioned respectively.

The figure below explains how each anti-body was cut from one blot.

As it is not possible to use 1 antibody per blot, each of them were cut to fit the anti-body and used in the experiment. The reason behind it was that, the amount of the sample was not sufficient. In addition to this, the amount of anti-body was also limited.

All the data are original and not manipulated.

Only the merge operation was performed using Photoshop.

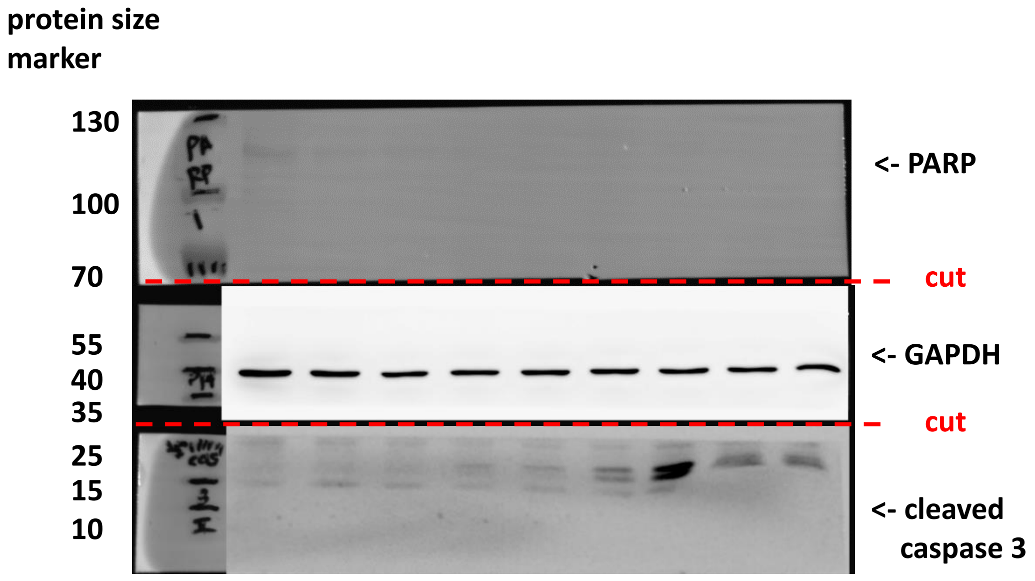

1. Figure-5; Cleaved Caspase 3-1 marker (merge)

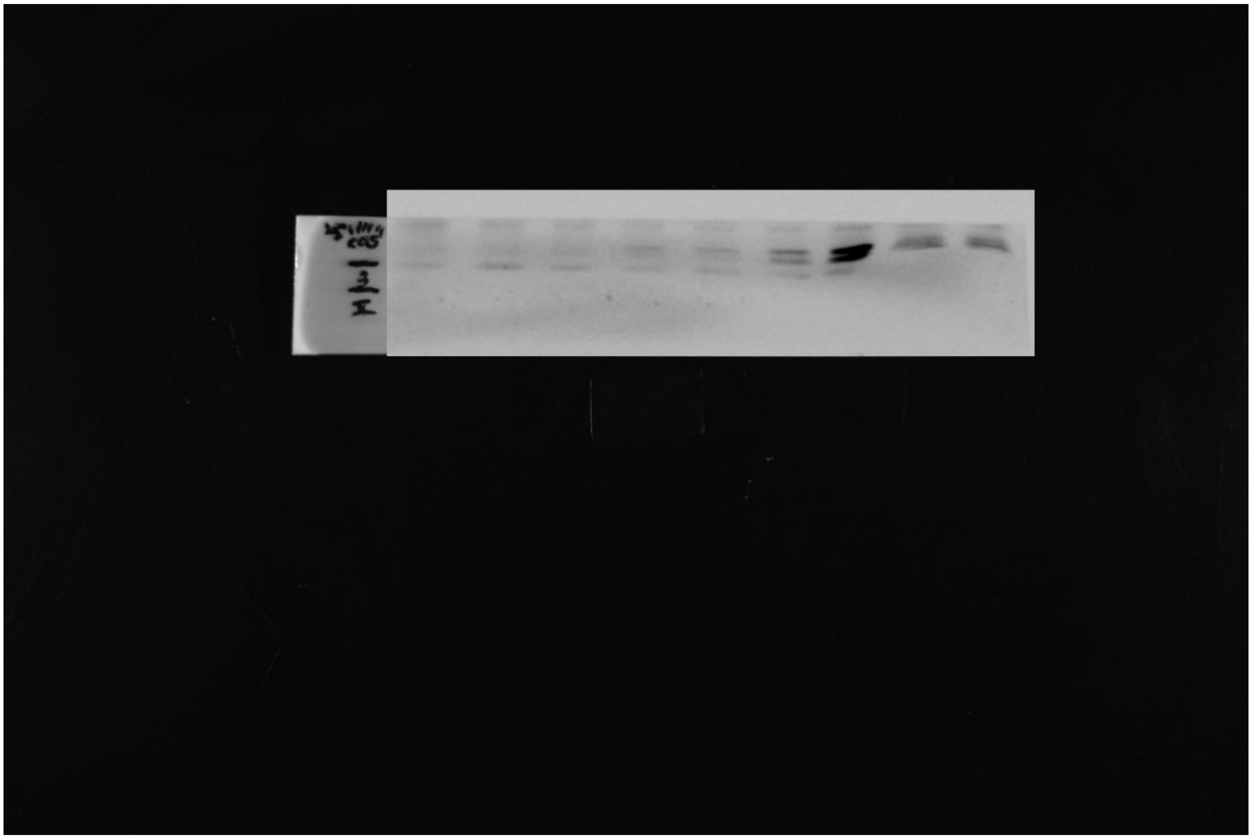

2. Figure 5; Cleaved Caspase 3-1(17,19 kDa)

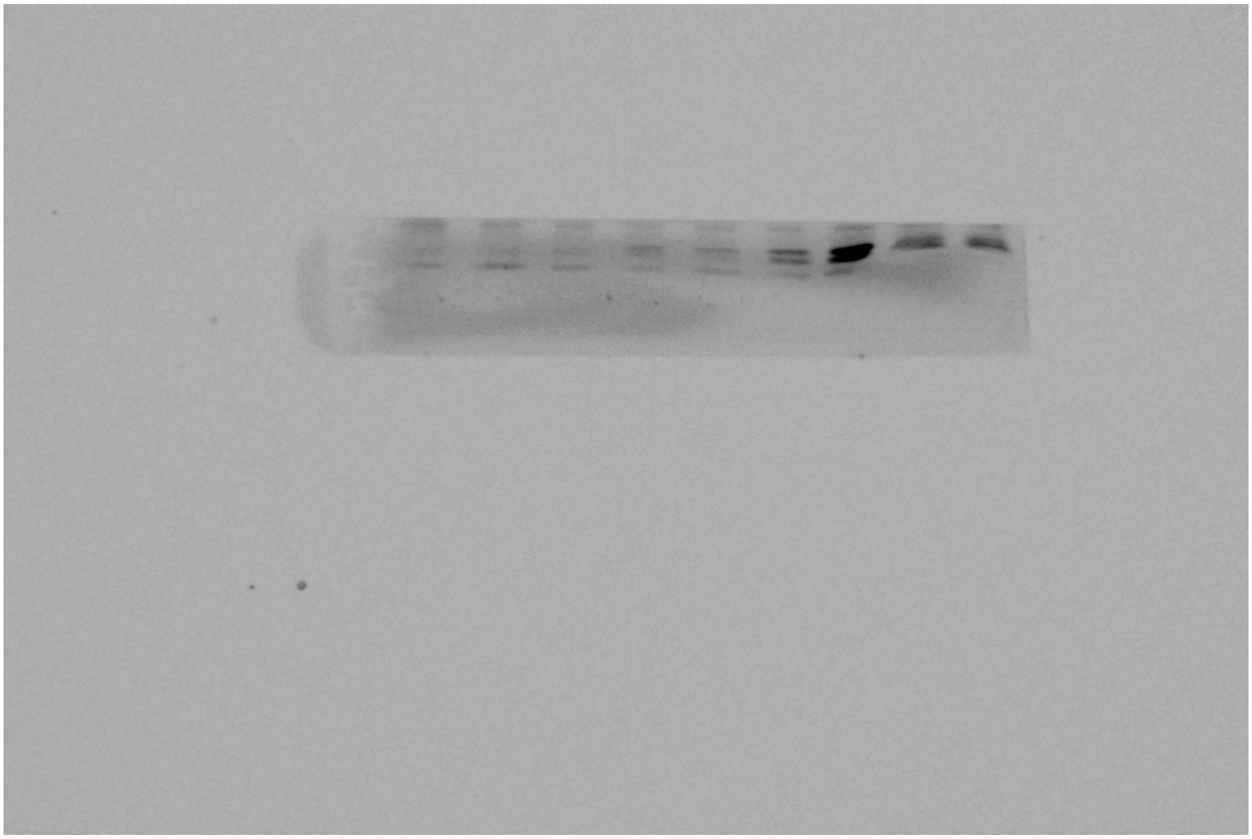

3. Figure-5; Cleaved Caspase 3-1 marker

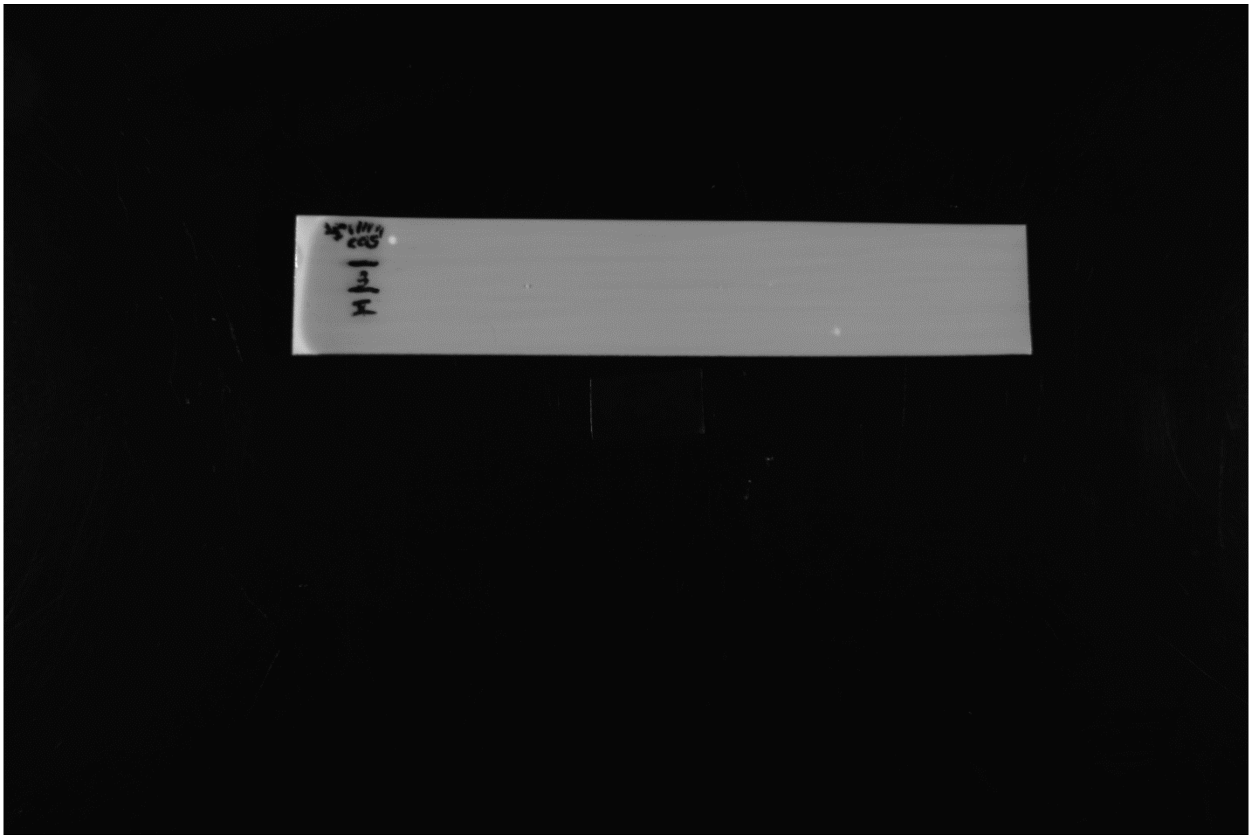

4. Figure-5; GAPDH-1 (37kDa)

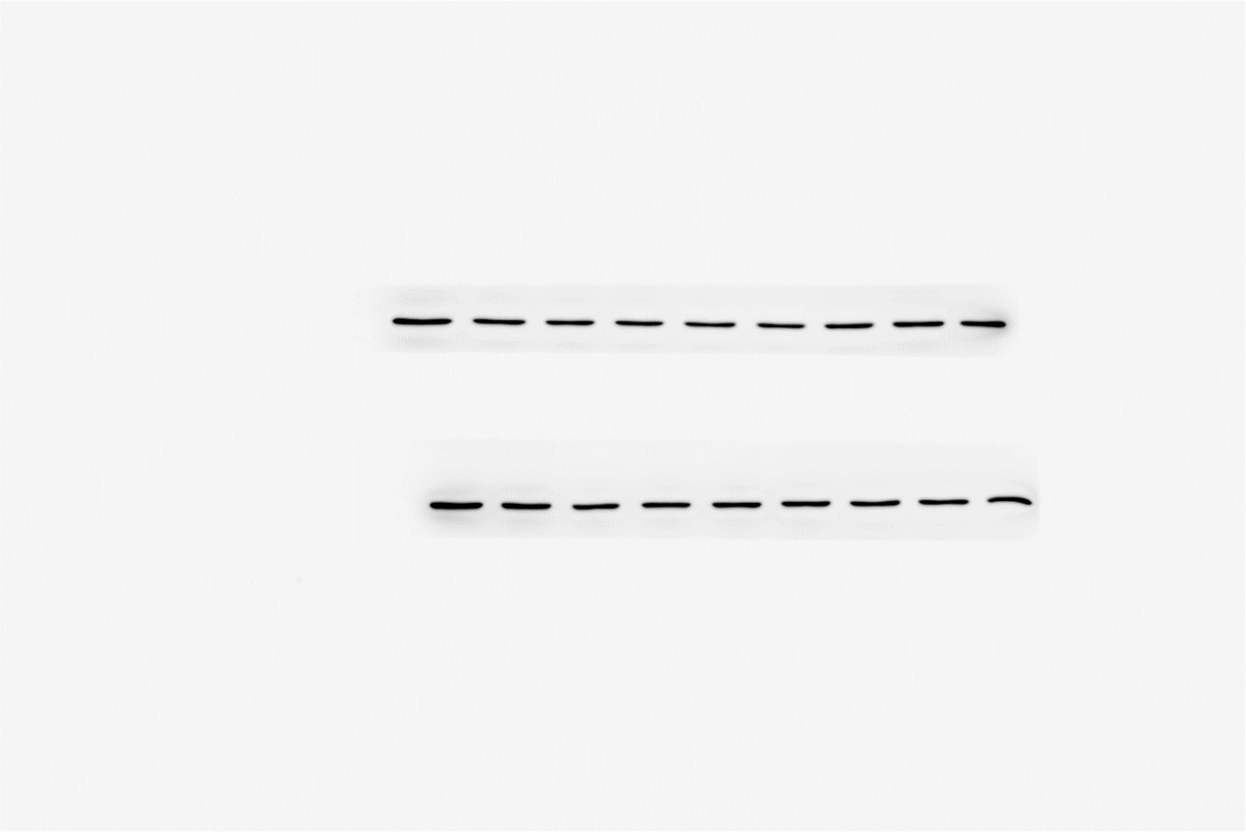

5. Figure-5; GAPDH-1 marker merge

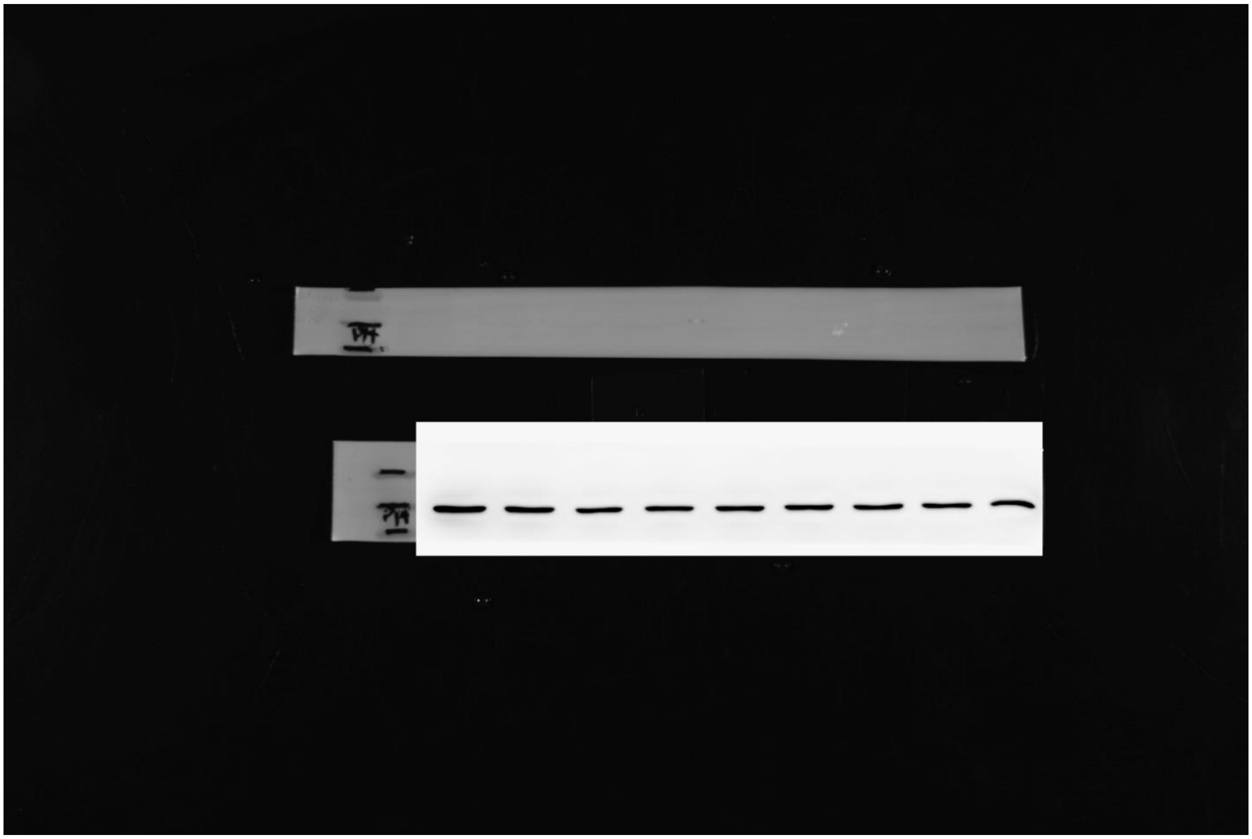

6. Figure-5; PARP-1 marker

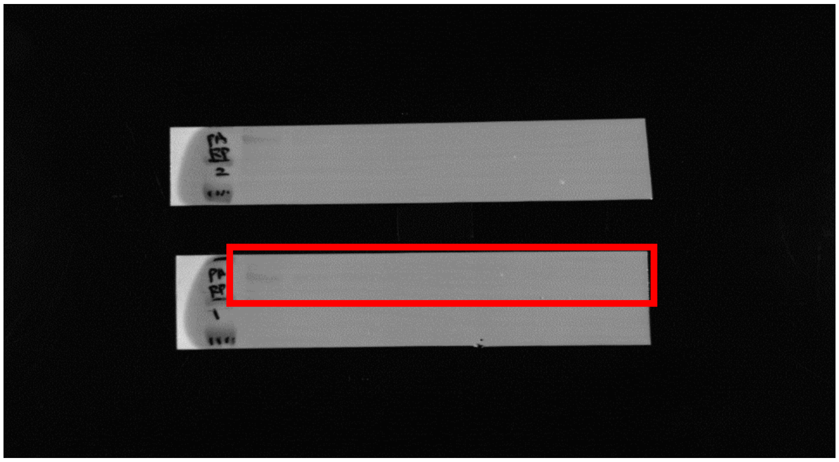

7. Figure-5; PARP-1(89,116kDa)

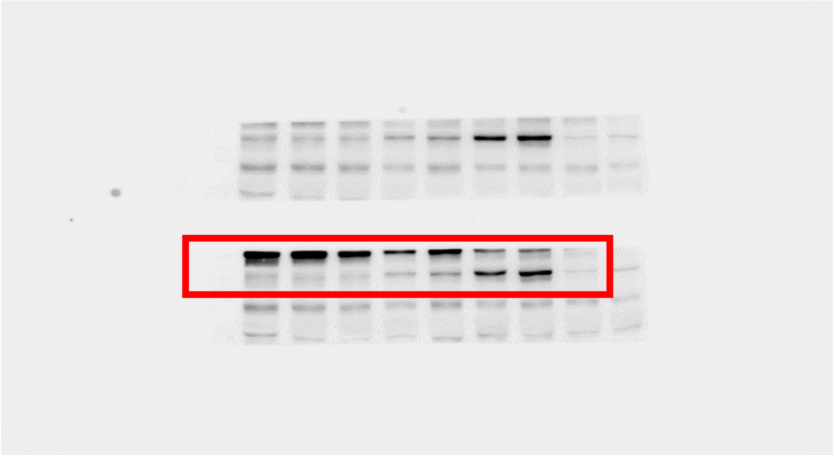

8. 3. Figure-5; PARP-1 marker merge

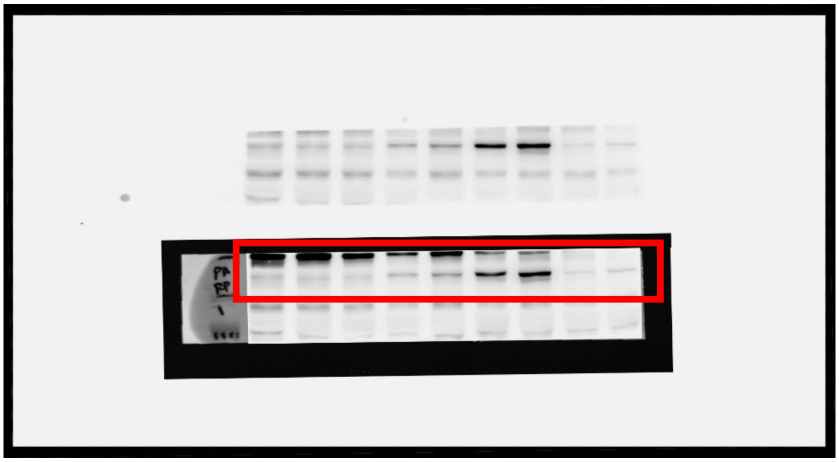

9. Figure-8; β-actin (42kDa) – upper blot

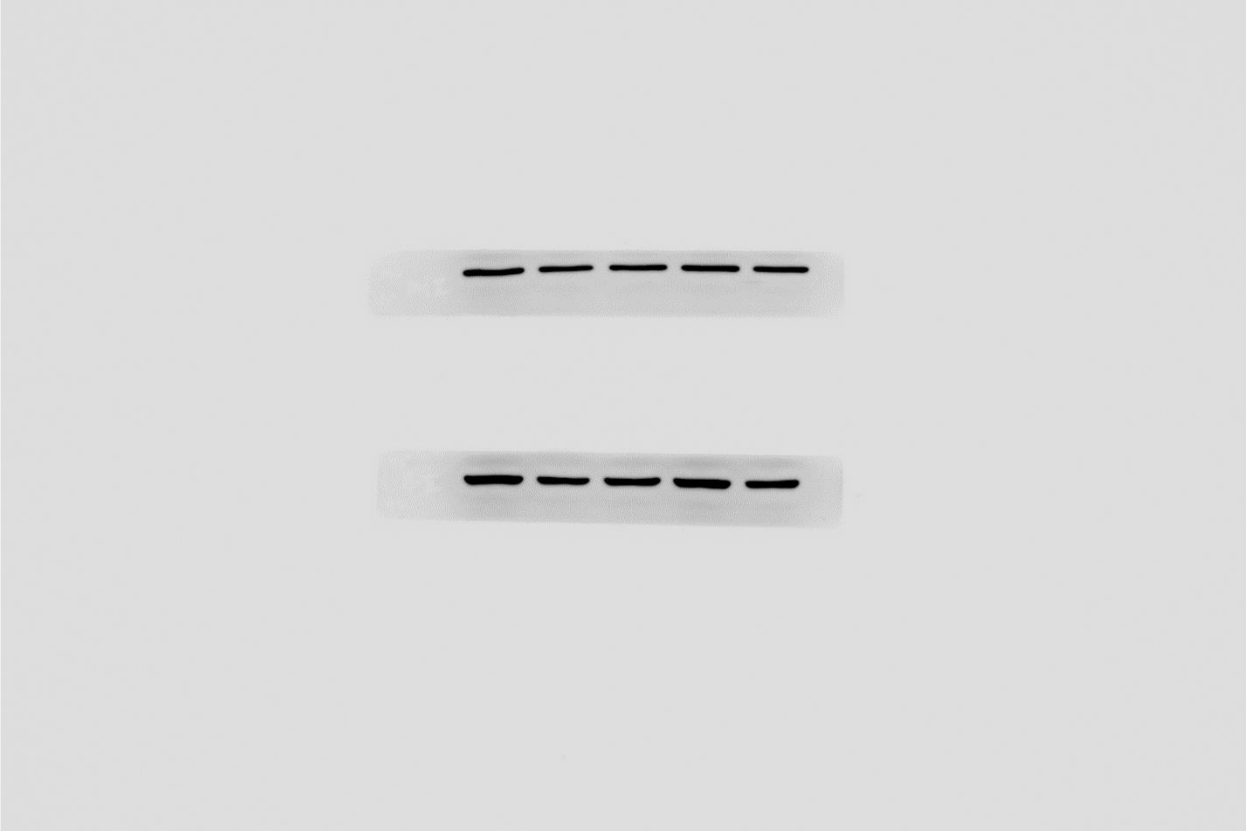

10. Figure-8; β-actin marker (upper blot)

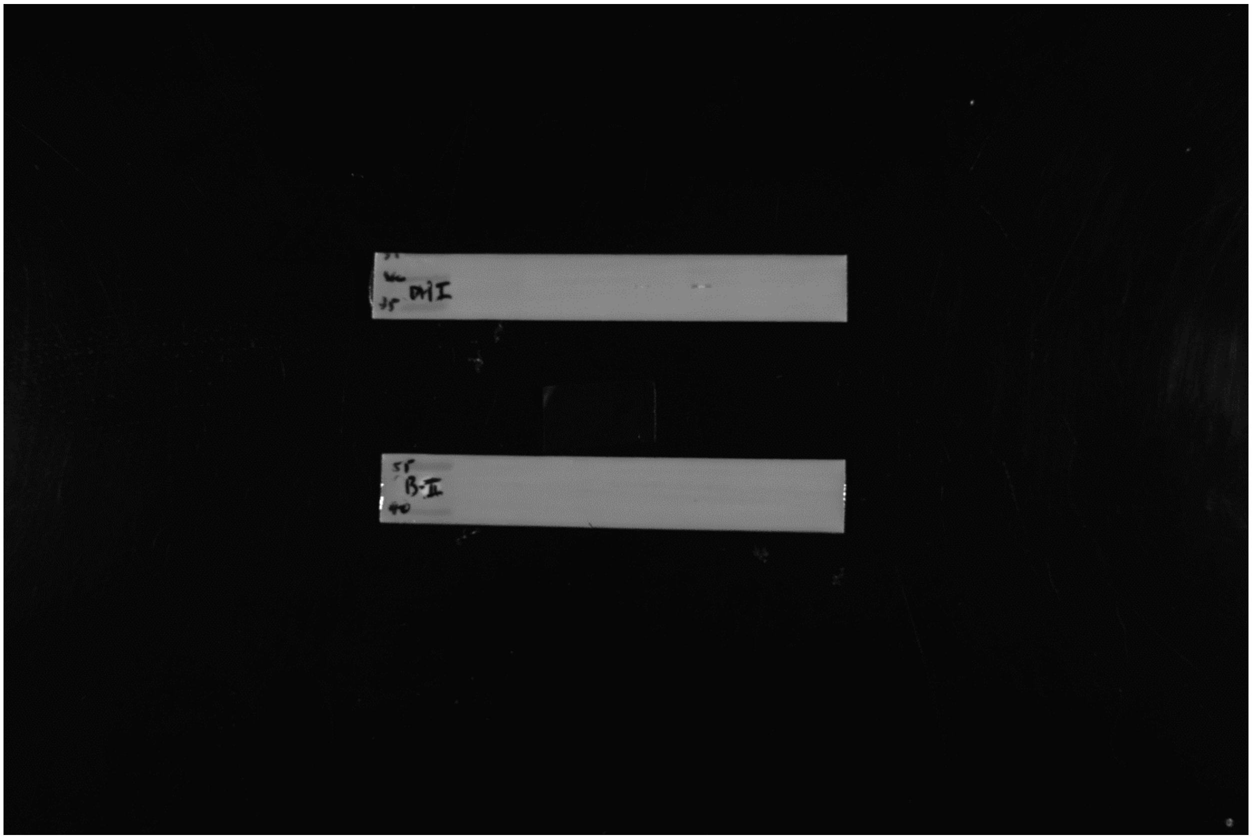

11. Figure-8; Cleaved Caspase 3 (17,19kDa)

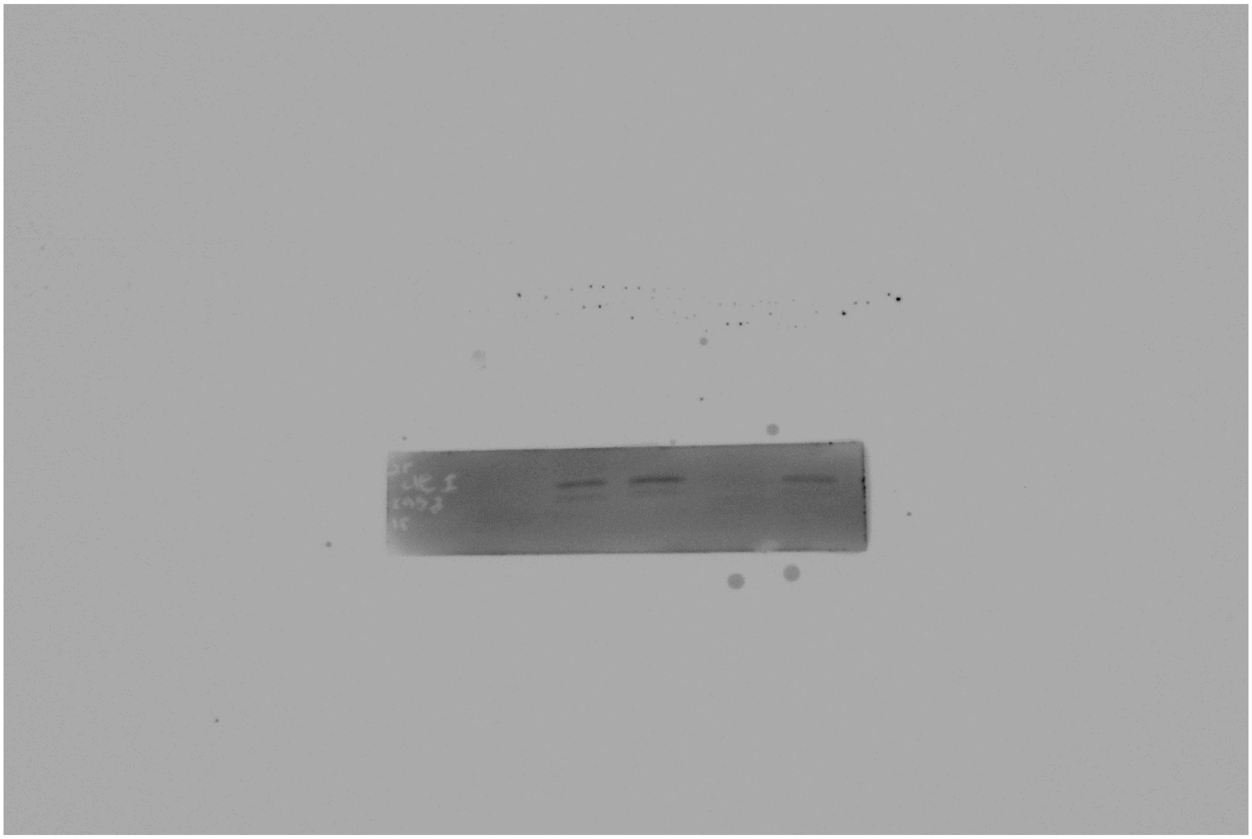

12. Figure-8; Cleaved Caspase 3 marker

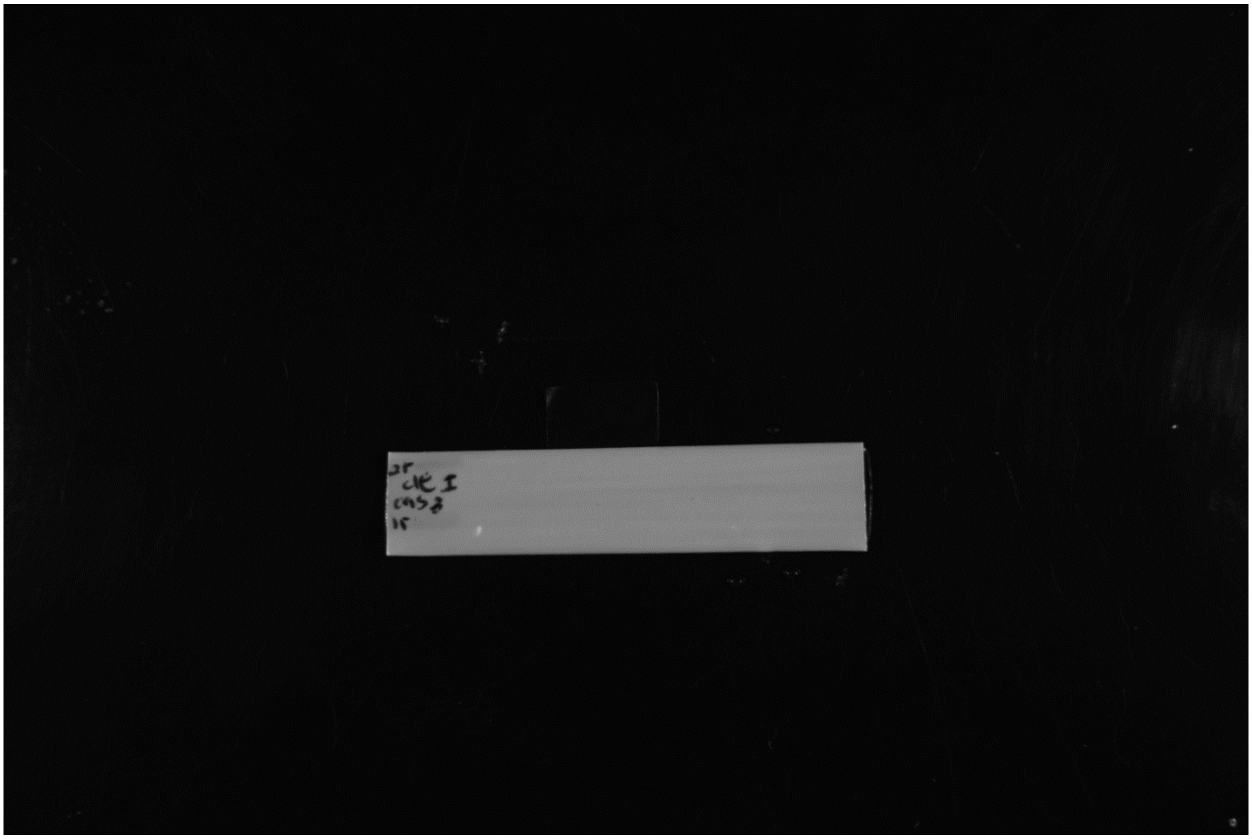

13. Figure-8; PARP 1 (89,116 kDa)

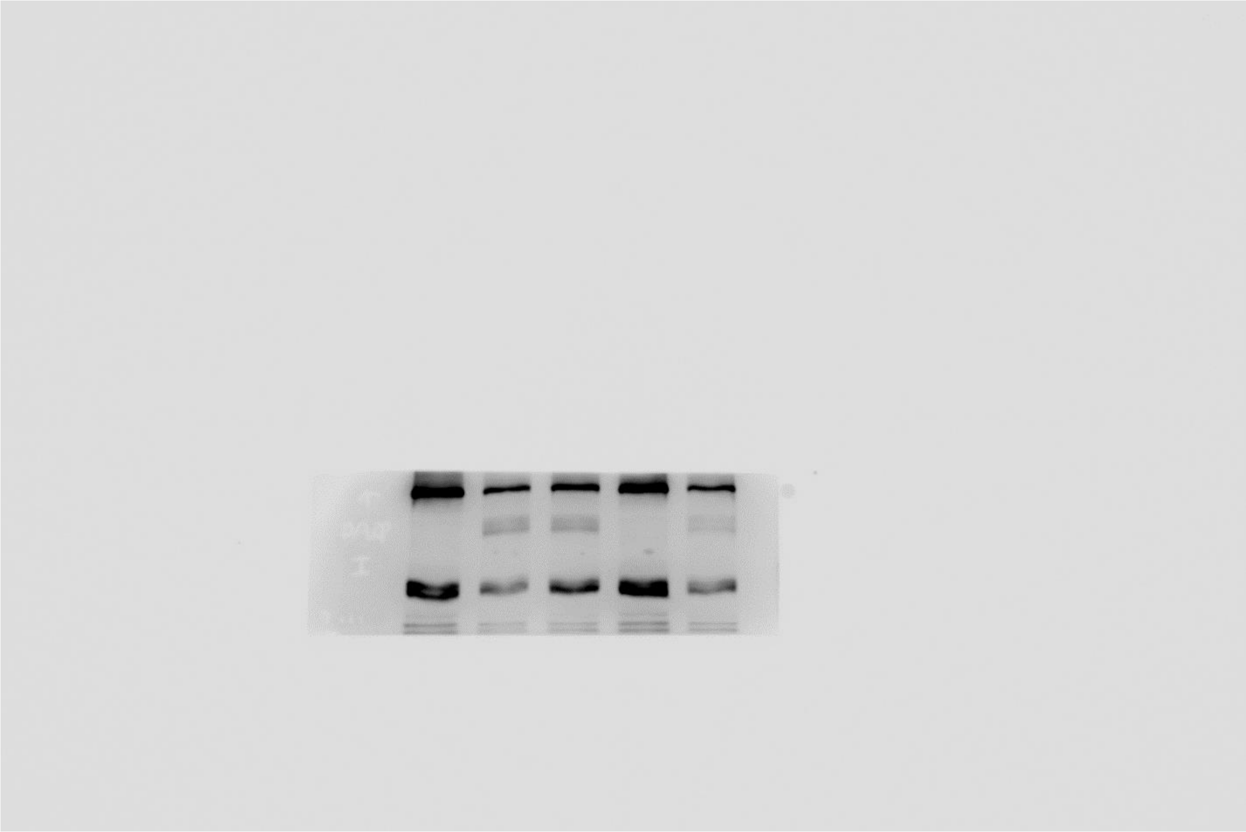

14. Figure-8; PARP 1 marker

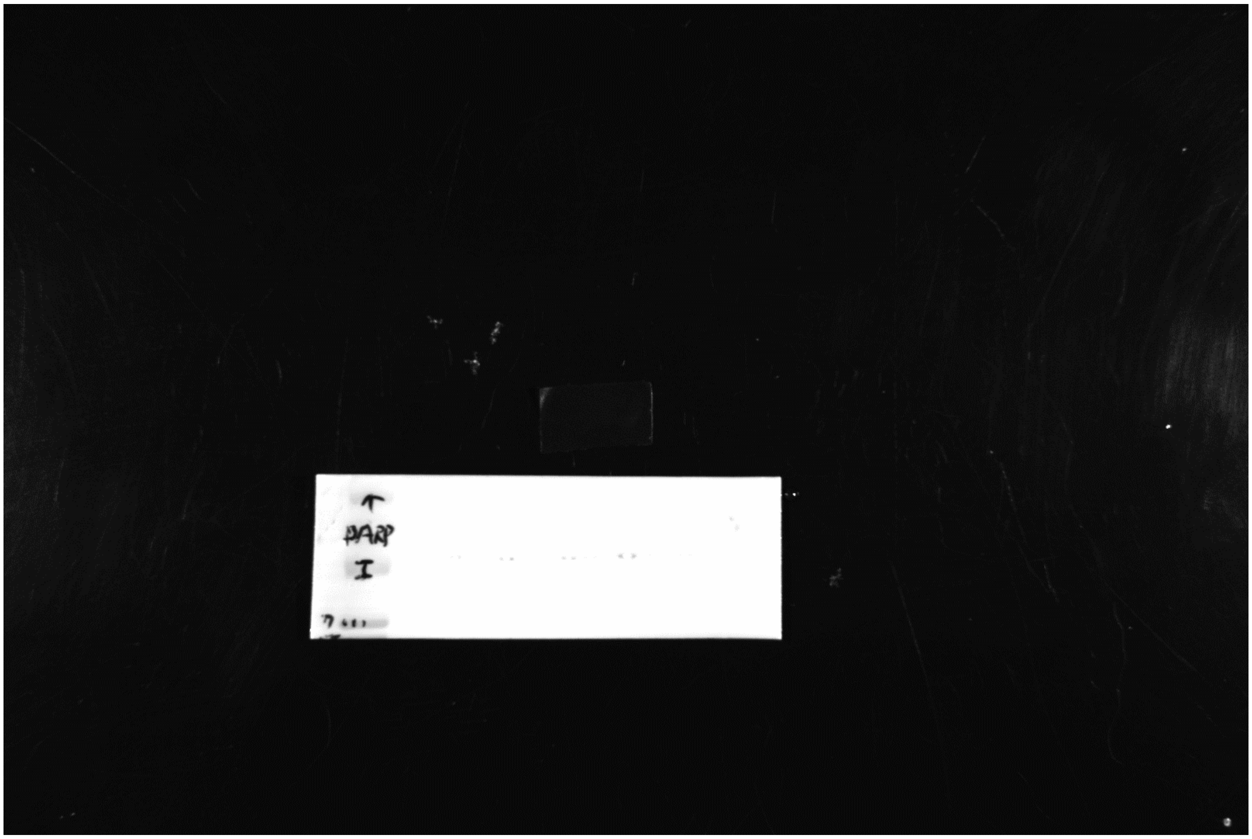

15. Figure-9; β-actin (42kDa) upper blot

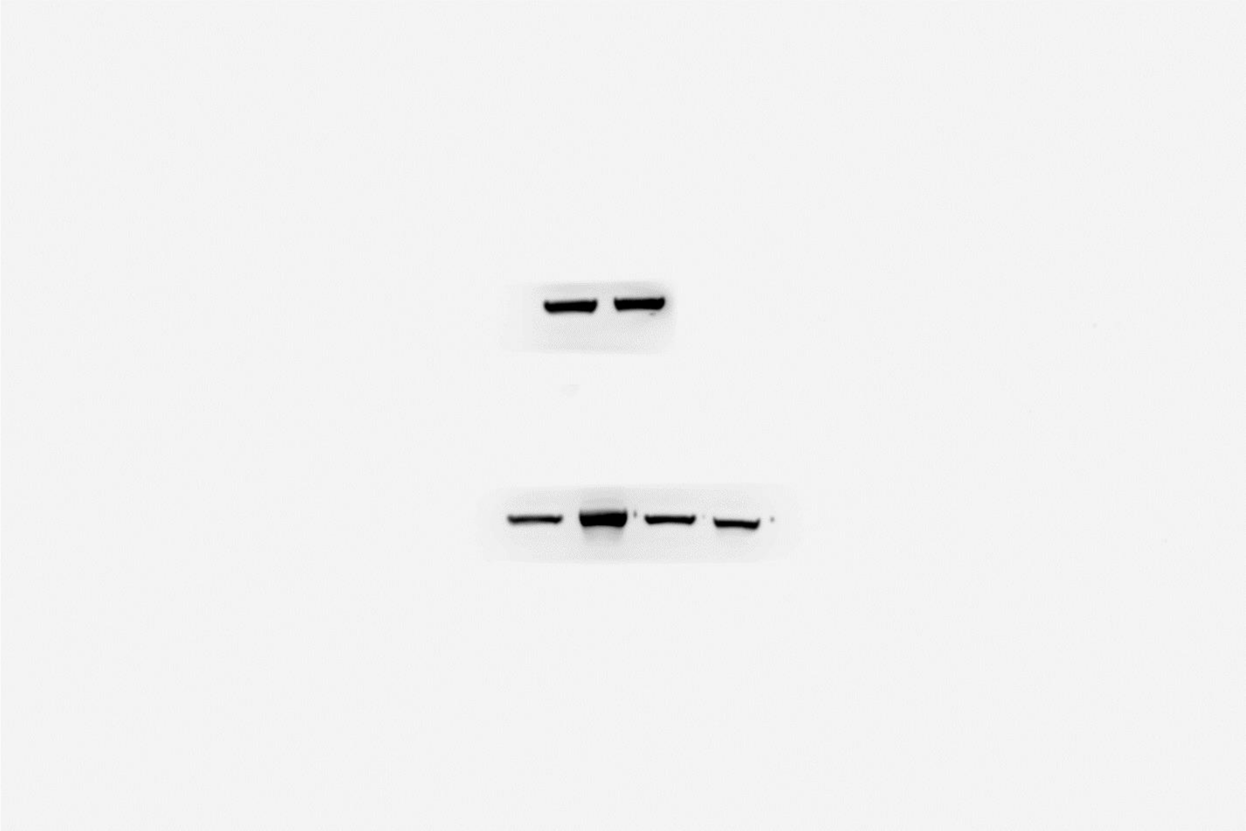

16. Figure-9; AQP 3 (36kDa)- upper blot

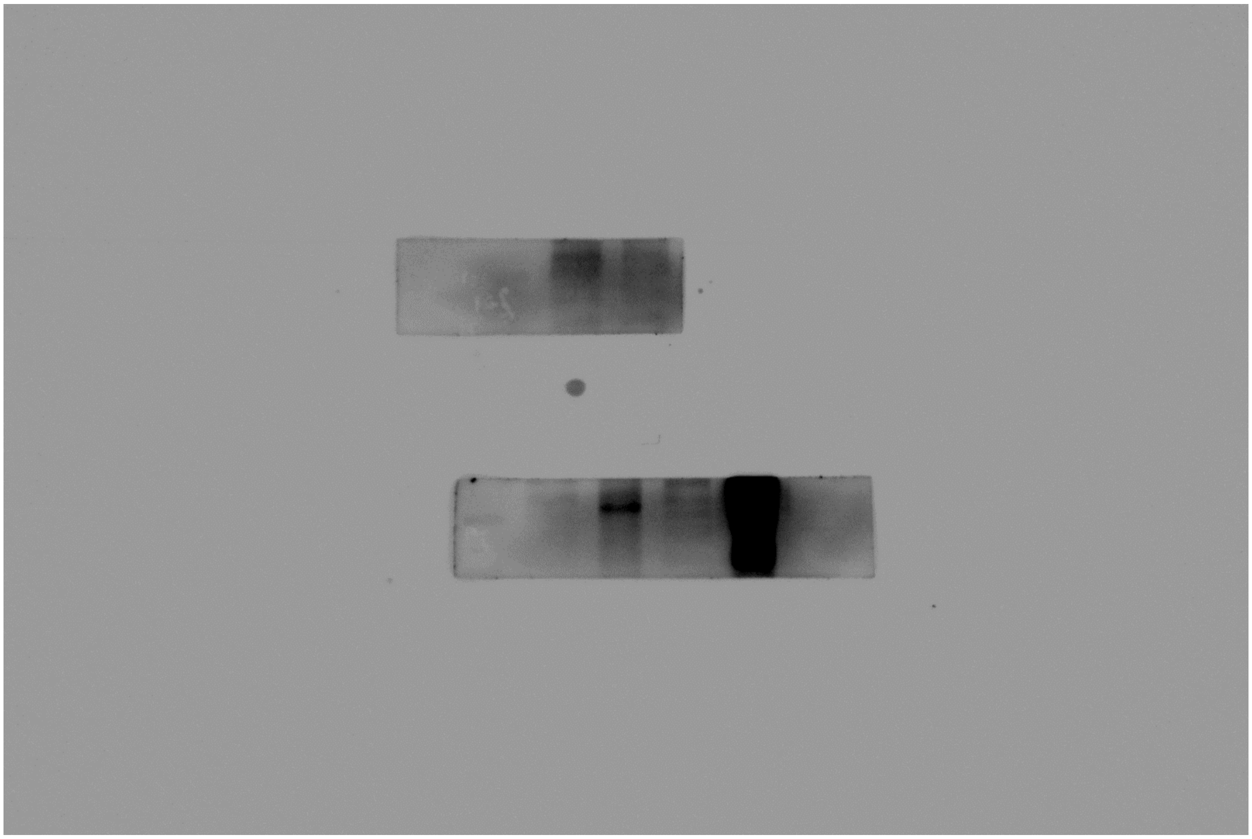

17. Figure-9; AQP 8 (34kDa)- upper blot

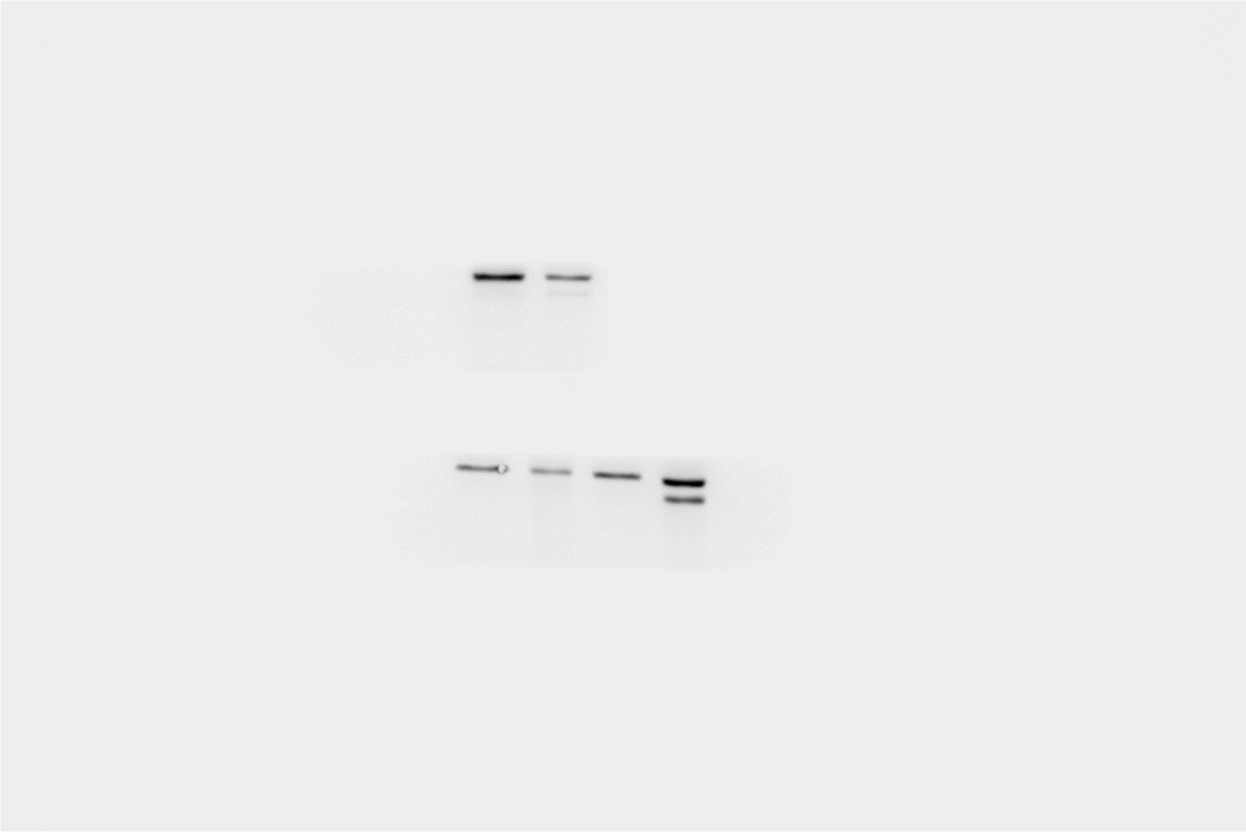

Supplement: Supplementary file 1 — Additional file 1. [file 12906_2023_4313_MOESM1_ESM.pdf]
